# Supplementary material for: Mixed-methods evaluation of a behavioural intervention package to identify and amend incorrect penicillin allergy records in UK general practice
Source: BMJ Open. 2022 Jun 3;12(6):e057471. doi: 10.1136/bmjopen-2021-057471 (PMC9171226; doi:10.1136/bmjopen-2021-057471)
Supplement: Supplementary data [file bmjopen-2021-057471supp001.pdf]

Appendices

Appendix 1 Clinician Penicillin Allergy Belief questionnaire

Please respond to the statements below using the scale provided. Please give only one answer per statement.

I understand what is involved in penicillin allergy testing.

☐	☐	☐	☐	☐	☐	☐
Strongly Agree	Agree	Slightly Agree	Not Sure	Slightly Disagree	Disagree	Strongly Disagree

I have previous experience of referring patients for penicillin allergy testing.

☐	☐	☐	☐	☐	☐	☐
Strongly Agree	Agree	Slightly Agree	Not Sure	Slightly Disagree	Disagree	Strongly Disagree

I am happy to refer patients for penicillin allergy testing.

☐	☐	☐	☐	☐	☐	☐
Strongly Agree	Agree	Slightly Agree	Not Sure	Slightly Disagree	Disagree	Strongly Disagree

Penicillin allergy testing can benefit my patients.

☐	☐	☐	☐	☐	☐	☐
Strongly Agree	Agree	Slightly Agree	Not Sure	Slightly Disagree	Disagree	Strongly Disagree

Penicillin allergy testing can benefit my practice.

Strongly Agree	Agree	Slightly Agree	Not Sure	Slightly Disagree	Disagree	Strongly Disagree
☐	☐	☐	☐	☐	☐	☐

It is safe to prescribe penicillin after a negative penicillin allergy test.

Strongly Agree	Agree	Slightly Agree	Not Sure	Slightly Disagree	Disagree	Strongly Disagree
☐	☐	☐	☐	☐	☐	☐

I am confident in discussing penicillin allergy test results with my patients.

Strongly Agree	Agree	Slightly Agree	Not Sure	Slightly Disagree	Disagree	Strongly Disagree
☐	☐	☐	☐	☐	☐	☐

I am happy to change patient records based on the results of penicillin allergy testing.

Strongly Agree	Agree	Slightly Agree	Not Sure	Slightly Disagree	Disagree	Strongly Disagree
☐	☐	☐	☐	☐	☐	☐

I would prescribe penicillin, if indicated, to patients with a negative penicillin allergy test result.

Strongly Agree	Agree	Slightly Agree	Not Sure	Slightly Disagree	Disagree	Strongly Disagree
☐	☐	☐	☐	☐	☐	☐

My colleagues support de-labelling of patients with incorrect penicillin allergy status using penicillin allergy testing.

Strongly Agree	Agree	Slightly Agree	Not Sure	Slightly Disagree	Disagree	Strongly Disagree
☐	☐	☐	☐	☐	☐	☐

My patients would be happy to be referred for penicillin allergy testing.

☐	☐	☐	☐	☐	☐	☐
Strongly Agree	Agree	Slightly Agree	Not Sure	Slightly Disagree	Disagree	Strongly Disagree

My patients would be happy to take penicillin following a negative penicillin allergy test.

☐	☐	☐	☐	☐	☐	☐
Strongly Agree	Agree	Slightly Agree	Not Sure	Slightly Disagree	Disagree	Strongly Disagree

Appendix 2 Patient Penicillin Allergy Belief questionnaire

Below statements to be asked to patients with responses given as Likert scale (1-7):

14. Penicillin is often the best treatment for bacterial infections.

☐	☐	☐	☐	☐	☐	☐
Strongly Agree	Agree	Slightly Agree	Not Sure	Slightly Disagree	Disagree	Strongly Disagree

15. Having an incorrect penicillin allergy label can be harmful for patients.

☐	☐	☐	☐	☐	☐	☐
Strongly Agree	Agree	Slightly Agree	Not Sure	Slightly Disagree	Disagree	Strongly Disagree

16. Having a penicillin allergy test is not beneficial for patients themselves.

☐	☐	☐	☐	☐	☐	☐
Strongly Agree	Agree	Slightly Agree	Not Sure	Slightly Disagree	Disagree	Strongly Disagree

17. Most people in the UK who think they are allergic to penicillin are not actually allergic.

☐ Strongly Agree ☐ Agree ☐ Slightly Agree ☐ Not Sure ☐ Slightly Disagree ☐ Disagree ☐ Strongly Disagree

18. I believe that I am allergic to penicillin.

☐ Strongly Agree ☐ Agree ☐ Slightly Agree ☐ Not Sure ☐ Slightly Disagree ☐ Disagree ☐ Strongly Disagree

19. There is a high chance that I would have a serious allergic reaction if I took penicillin.

☐ Strongly Agree ☐ Agree ☐ Slightly Agree ☐ Not Sure ☐ Slightly Disagree ☐ Disagree ☐ Strongly Disagree

20. It is important for me to know whether or not I am allergic to penicillin.

☐ Strongly Agree ☐ Agree ☐ Slightly Agree ☐ Not Sure ☐ Slightly Disagree ☐ Disagree ☐ Strongly Disagree

21. Penicillin allergy testing is safe.

☐ Strongly Agree ☐ Agree ☐ Slightly Agree ☐ Not Sure ☐ Slightly Disagree ☐ Disagree ☐ Strongly Disagree

22. Penicillin allergy testing can say if someone is allergic to penicillin or not.

☐ Strongly Agree ☐ Agree ☐ Slightly Agree ☐ Not Sure ☐ Slightly Disagree ☐ Disagree ☐ Strongly Disagree

23. I would benefit from being able to take penicillin antibiotics safely when needed in future.

☐ Strongly Agree ☐ Agree ☐ Slightly Agree ☐ Not Sure ☐ Slightly Disagree ☐ Disagree ☐ Strongly Disagree

24. I am frightened about having a serious allergic reaction if I take penicillin.

☐ Strongly Agree ☐ Agree ☐ Slightly Agree ☐ Not Sure ☐ Slightly Disagree ☐ Disagree ☐ Strongly Disagree

25. My doctor would be happy to prescribe penicillin if a test showed I was not allergic to penicillin.

☐ Strongly Agree ☐ Agree ☐ Slightly Agree ☐ Not Sure ☐ Slightly Disagree ☐ Disagree ☐ Strongly Disagree

26. I would be happy to take penicillin prescribed by my doctor if a test showed I was not allergic.

☐ Strongly Agree ☐ Agree ☐ Slightly Agree ☐ Not Sure ☐ Slightly Disagree ☐ Disagree ☐ Strongly Disagree

Appendix 3

Clinician Topic Guide

Below is a list of topics to be discussed in this study. The work will remain flexible with respect to participants' agendas. Therefore, we may add new topics as the interviews progress and data collection continues. However, the key topic of clinicians' views and experiences of managing patients with penicillin allergy and participation in the ALABAMA trial will remain the same.

1. Participants' views and experiences of referring patients for penicillin allergy testing (e.g. views of the test, identification of eligible patients)
2. Participants' views and experiences of prescribing antibiotics following a negative allergy test result (e.g. views on prescribing penicillin, discussing test results with patients)

3. Participants' views and experiences of taking part in the trial (e.g. process of recruiting patients, views of trials procedures, views of the trial documents)
4. Participants' views and experiences of intervention materials (e.g. ease of understanding, helpfulness of the documents)

Example questions (additional questions may be added during interviews following the topics above):

The ALABAMA study:

1. Could you tell me why your practice signed up to the ALABAMA study?
   Prompts: Why was the topic of interest? Who was responsible for joining the study? How/when did you first hear about the study?
2. Could you tell me how you found taking part in the ALABAMA study?
   Prompts: To what extent did you feel informed about the study? Did you have any concerns about inviting patients to the study? How did you find the site training? Was there anything you would add or change to the training you received?

Penicillin allergy testing:

3. What do you think about incorrect penicillin allergy records within general practice?
   Prompts: What impact do incorrect allergy records have on patient care? How confident are you about your knowledge of allergies and penicillin allergy testing? Do you have any concerns about allergy testing?
4. How did you feel about referring patients for penicillin allergy testing as part of the trial?
   Prompts: If you referred patients as part of the trial, how did you find the referral of patients? How confident were you in deciding which patients could be referred for a test? Have you encountered any barriers to referral? How did you overcome them? How did you find the Working Instructions on screening and referral of patients? Have you encountered any barriers while using them? Were they easy to find? Were they easy to read? Is there anything you would change about them?
5. How did you find the consultations with patients when they came to be consented for the trial?
   Prompts: What were the most common questions and concerns raised by patients? How confident were you in addressing patients' questions and concerns? Did you use the Working Instructions for these consultations? Have you experienced any barriers when using these

working instructions? Were they easy to find? Were they easy to read? Is there anything about them that you would change?

6. What did you think about the trial materials that you received during the trial?
   Prompts: Was the information you received helpful? Is there anything that should be added to or changed?

7. You received an information pack to help you manage patients who had had a negative allergy test. What did you think of this document?
   Prompts: How were you given the document? Was it helpful? How easy or difficult was it to follow? Is there anything that should be added to or changed? How did you use the Information pack? What did you think of the electronic pop-up about the test results? Is there anything you would change or add to the electronic pop-up?

8. Patients also received information booklets on penicillin allergy testing as part of the trial. What did you think about the patient information provided?
   Prompts: Did you discuss the information with patients in consultations after the test? How did you use the booklet? Is there anything that you would change or add to the patient booklet? Has any patient used the post-intervention card during a consultation about infections after the test? If so, what did you think about the card?

9. How did you receive the allergy test results for your patients?
   Prompts: What did you think about the letters that you received with the patients' test results? Is there anything that you would change or add to that letter? What did you do when the test came back negative/positive? How confident were you in the results? Did you discuss results with the patient? If so when did this happen and how did you explain the results?

10. What was the process of updating medical records?
    Prompts: Have you updated the medical records following negative test result letters? Did you use the working instructions on updating the medical records? How did you find them? Were they easy to find? Were they easy to read? Is there anything that you would change about them? Have you experienced any barriers in updating medical records? How did you overcome them? What did you think of the information about how to update medical records?

11. To what extent did the test result change the way you manage patients now?
    Prompts: Have any patients with infections consulted you following the test? If so, what happened? Have you prescribed penicillin to any patient who had a negative test who has

presented with an infection which needed antibiotics? If so, what did the patient(s) think about taking penicillin?

12. How would you feel about having extended penicillin allergy testing available in the long-term? Prompts: How do you think you would refer to the service? Who do you think it would benefit? Would you have any concerns about continuing to refer patients?

13. Do you have any other thoughts about how the intervention went?

Patient Topic Guide

Below is a list of topics to be discussed in this study. The work will remain flexible with respect to participants' agendas. Therefore, we may add new topics as the interviews progress and data collection continues. However, the key topic of patients' views and experiences of penicillin allergy testing and participation in the trial will remain the same.

1. Participants' views and experiences of undergoing penicillin allergy testing (e.g. what it was like, barriers and facilitators to attending, views about the results, views on taking penicillin when prescribed first line if allergy test was negative)
2. Participants' views and experiences of taking part in the trial (e.g. process of making decisions to take part, views of trials procedures, barriers and facilitators to taking part, views of the trial documents)
3. Participants' views and experiences of intervention materials (ease of understanding, helpfulness of the documents)

Example questions (additional questions may be added following the topics above):

The AlABAMA study:

1. Can you tell me about why you decided to take part in the AlABAMA study? Prompts: What did you think when you first heard about the study? Who did you discuss the study with before deciding to take part?

2. What information were you given about the study? Prompts: What did you think of the letter and information you were sent about the study?

3. How helpful was this information?
4. Did you have any concerns about taking part?

Penicillin allergy testing:

5. What did you know about penicillin allergy before taking part in the study?
   Prompts: What did you think about having a penicillin allergy? How much did you know about the benefits of taking penicillin compared to other types of antibiotics? How confident are you about what you know now about it? How much did you know about incorrect penicillin allergy labels? How confident are you about what you know now about it?

6. What did you know about penicillin allergy testing before the trial?
   Prompts: What did you think the test was going to involve? What concerns did you have about the test? What do you think about the appointment you had at your general practice before you took the test?

   Prompt: How useful was the appointment? Could you tell me more about any questions or concerns that you discussed during the appointment? How did the GP/nurse answer your questions and concerns?

[For patients in the PAAP intervention arm]

7. Can you tell me what happened when you went for the test in the allergy clinic?
   Prompts: What information were you given before the test? How did you feel about going for the test? Were there any barriers to attending the test? Which health professionals did you speak to about the test? Were there any difficulties in completing the test at the hospital? ow safe did you feel during the test?

8. Were there any difficulties in completing the test at home?
   Prompts: What barriers did you encounter in completing the test at home? How did you overcome them?

9. What happened after your hospital visit?
   Prompts: What were you told to do following the test? Who did you talk to about the test? How confident were in knowing who to contact in case of a reaction at home?

10. How did you feel about the result?
    Prompts: Who did you discuss the result with?

11. [If negative test result] How do you feel about taking penicillin now?
    Prompts: What would you do if your GP wanted to prescribe you an antibiotic? Have you/will you discuss the test result with your GP? Have you been prescribed penicillin? If so, what did you think about taking penicillin?

[For patients in the PAAP intervention arm: Intervention materials]

12. As part of the trial you were provided with a booklet on penicillin allergy testing. What did you think of this booklet?
    Prompts: Was the booklet helpful? How did you use the booklet? Is there anything you would add or change in the booklet?

13. [For patients with negative test result] When you were sent the allergy test result you were given another booklet and a card. What did you think of these?
    Prompts: Was the booklet/card helpful? How did you use the booklet? Do you still have the booklet? Would you use the card in future? Have you used the card with your GP or nurse? Is there anything you would add or change in the booklet and card?

14. Do you have any other thoughts about how the study went? What changes would you recommend for the future trial?
