# Supplementary material for: Mixed-methods evaluation of a behavioural intervention package to identify and amend incorrect penicillin allergy records in UK general practice
Source: BMJ Open. 2022 Jun 3;12(6):e057471. doi: 10.1136/bmjopen-2021-057471 (PMC9171226; doi:10.1136/bmjopen-2021-057471)
Supplement: Author's manuscript [file bmjopen-2021-057471.draft_revisions.pdf]

BMJ Open is committed to open peer review. As part of this commitment we make the peer review history of every article we publish publicly available.

When an article is published we post the peer reviewers' comments and the authors' responses online. We also post the versions of the paper that were used during peer review. These are the versions that the peer review comments apply to.

The versions of the paper that follow are the versions that were submitted during the peer review process. They are not the versions of record or the final published versions. They should not be cited or distributed as the published version of this manuscript.

BMJ Open is an open access journal and the full, final, typeset and author-corrected version of record of the manuscript is available on our site with no access controls, subscription charges or pay-per-view fees (http://bmjopen.bmj.com).

If you have any questions on BMJ Open's open peer review process please email
info.bmjopen@bmj.com

BMJ Open

Mixed-methods evaluation of a behavioural intervention package to identify and amend incorrect penicillin allergy records in UK general practice

Journal:	*BMJ Open*
Manuscript ID	bmjopen-2021-057471
Article Type:	Original research
Date Submitted by the Author:	16-Sep-2021

Complete List of Authors:	Wanat, Marta; Oxford University, Nuffield Department of Primary Care Health Sciences<br>Santillo, Marta; Oxford University, Nuffield Department of Primary Care Health Sciences<br>Galal, Ushma; University of Oxford, Nuffield Department of Primary Care Health Sciences<br>Davoudianfar, Mina; University of Oxford, Nuffield Department of Primary Care Health Sciences<br>Bongard, Emily; University of Oxford, Nuffield Department of Primary Care Health Sciences<br>Savic, Sinisa; University of Leeds Faculty of Medicine and Health,<br>Savic, Louise; University of Leeds Faculty of Medicine and Health<br>Porter, Catherine; University of Leeds, Healthcare Associated Infection Group<br>Fielding, Joanne; University of Leeds, Healthcare Associated Infection Group<br>Butler, Christopher C.; University of Oxford, Nuffield Department of Primary Health Care Sciences<br>Pavitt, Sue; University of Leeds, Dental Translational and Clinical Research Unit, Faculty of Medicine and Health,<br>Sandoe, Jonathan; University of Leeds Faculty of Medicine and Health, Healthcare Associated Infection Group<br>Tonkin-Crine, Sarah ; University of Oxford, Nuffield Department of Primary Care Health Science

Keywords:	PRIMARY CARE, IMMUNOLOGY, QUALITATIVE RESEARCH

SCHOLARONE™
Manuscripts

I, the Submitting Author has the right to grant and does grant on behalf of all authors of the Work (as defined in the below author licence), an exclusive licence and/or a non-exclusive licence for contributions from authors who are: i) UK Crown employees; ii) where BMJ has agreed a CC-BY licence shall apply, and/or iii) in accordance with the terms applicable for US Federal Government officers or employees acting as part of their official duties; on a worldwide, perpetual, irrevocable, royalty-free basis to BMJ Publishing Group Ltd ("BMJ") its licensees and where the relevant Journal is co-owned by BMJ to the co-owners of the Journal, to publish the Work in this journal and any other BMJ products and to exploit all rights, as set out in our licence.

The Submitting Author accepts and understands that any supply made under these terms is made by BMJ to the Submitting Author unless you are acting as an employee on behalf of your employer or a postgraduate student of an affiliated institution which is paying any applicable article publishing charge ("APC") for Open Access articles. Where the Submitting Author wishes to make the Work available on an Open Access basis (and intends to pay the relevant APC), the terms of reuse of such Open Access shall be governed by a Creative Commons licence – details of these licences and which Creative Commons licence will apply to this Work are set out in our licence referred to above.

Other than as permitted in any relevant BMJ Author's Self Archiving Policies, I confirm this Work has not been accepted for publication elsewhere, is not being considered for publication elsewhere and does not duplicate material already published. I confirm all authors consent to publication of this Work and authorise the granting of this licence.

For peer review only

Mixed-methods evaluation of a behavioural intervention package to identify and amend incorrect penicillin allergy records in UK general practice

Wanat, M.* [1], Santillo, M.* [1], Galal, U. [1], Davoudianfar, M. [1], Bongard, E. [1], Savic, S. [2], Savic, L. [2], Porter, C. E. [3], Fielding, J. [3], Butler C. [1], Pavitt, S. H. [4], Sandoe, J. A. T. [3$], Tonkin-Crine, S. [1,5$] On behalf of the ALABAMA team.

*Joint first authors

$Joint senior authors

Corresponding author: Dr Marta Wanat, Nuffield Department of Primary Care Health Sciences, University of Oxford, Radcliffe Observatory Quarter, Woodstock Road, Oxford, OX2 6GG , UK; email: marta.wanat@phc.ox.ac.uk

[1] Nuffield Department of Primary Care Health Sciences, University of Oxford, Radcliffe Observatory Quarter, Woodstock Road, Oxford, OX2 6GG , UK

[2] Faculty of Medicine and Health, University of Leeds and Leeds Teaching Hospitals NHS Trust, Leeds LS1 3EX, UK

[3] Healthcare Associated Infection Group, University of Leeds and Leeds Teaching Hospitals NHS Trust, Leeds LS1 3EX, UK

[4] Dental Translational and Clinical Research Unit, Faculty of Medicine and Health, Worsley Building, Clarendon Way, University of Leeds, LS2 9LU, UK

[5] NIHR Health Protection Research Unit in Healthcare Associated Infections and Antimicrobial Resistance, University of Oxford, UK

For peer review only - http://bmjopen.bmj.com/site/about/guidelines.xhtml

Abstract

Objectives About 6% of the UK general practice population has a record of a penicillin allergy but fewer than 10% of these are likely to be truly allergic. The ALABAMA (Allergy Antibiotics and Microbial resistance) feasibility trial, randomised primary care patients with penicillin allergy to penicillin allergy assessment pathway (PAAP) or usual care to assess the effect on health outcomes. A behavioural intervention package was developed to aid de-labelling. This study aimed to investigate patients' and clinicians' views of Penicillin Allergy Testing (PAT).

Design We conducted a mixed-methods process evaluation which included a clinician survey, a patient survey (at baseline and follow up) and semi-structured interviews with patients and clinicians.

Settings The study was conducted in primary care.

Participants Patients and primary care clinicians.

Results Clinicians (N=53) were positive about PAT and its potential value but did not have previous experience of referring patients for a PAT and were unsure whether patients would take penicillin after a negative allergy test. Patients (N=38) were unsure whether they were severely allergic to penicillin and did not fear a severe allergic reaction to penicillin. Clinician interviews showed that they were already aware of the benefit of PAT. Interviews with patients suggested the importance of safety as patients valued having numerous opportunities to address their concerns about safety of the test.

Conclusions This study highlights the positive effects of the ALABAMA behavioural intervention for both patients and clinicians.

Keywords: primary care, immunology, qualitative research

For peer review only - http://bmjopen.bmj.com/site/about/guidelines.xhtml

Strengths and limitations of this study

- The study benefits from using both survey and interviews, providing complementary perspectives.
- The study would have benefitted from a follow-up survey with clinicians, which was not possible due to study delays
- The study recruited from one region in England and includes small numbers due to issues with recruitment and testing availability; hence the results should be extrapolated with caution.
- Not all trial participants completed the survey, meaning that only views of participants who chose to complete the survey are represented.
- Both patient and PCP interview participants were a convenience sample recruited within a feasibility study, hence not allowing to capture perspectives of patients who did not want to take part in the interviews.

Introduction

Six to 10% of primary care patients in the UK have a penicillin allergy record[1,2] but fewer than 10% of these patients are likely to be allergic when formally tested,[3] which means that a large proportion of these people are unnecessarily avoiding first-line antibiotic treatments. Penicillin allergy testing (PAT), usually undertaken in specialist immunology clinics, offers the opportunity to confirm or discount a penicillin allergy, although such services are only currently available for a limited number of patients. The negative consequences of having a penicillin allergy record include: suboptimal clinical outcomes, increased antibiotic resistance[4-6] and higher healthcare costs.[5,7,8]

Barriers to removing incorrect penicillin allergy records include lack of standardised protocols across practices,[9, 10] lack of access to skin testing,[11] and the difficulty of convincing patients that the record is no longer needed after a negative test.[12,13] These studies also suggest multiple ways to improve

For peer review only

research, testing and implementation of penicillin allergy de-labelling initiatives such as easier access to skin testing reagents, standardised skin testing protocols and a greater understanding of penicillin allergy epidemiology.

There is limited understanding of the views of patients and primary care healthcare professionals on de-labelling incorrect penicillin allergy records in primary care. The NIHR-funded ALABAMA programme includes a randomised controlled trial of primary care patients who are randomised to a pre-emptive Penicillin Allergy Assessment pathway (PAAP) or usual clinical care to assess the effect on health outcomes.

Within this study, we addressed the current gap, by investigating patient and clinician views and experiences of penicillin allergy testing as part of the mixed-methods process evaluation [14,15] of the ALABAMA feasibility trial. Our mixed-methods process evaluation included a clinician questionnaire, a patient questionnaire, and interviews with clinicians and patients.

Methods

The ALABAMA feasibility trial involved 11 general practices based in the Leeds/Bradford area of England. The behavioural intervention package was designed to target both general practice clinicians responsible for the prescription of antibiotics and patients with a suspected incorrect penicillin allergy record, and was an integral part of the PAAP. The details on the process, methods and outcomes of the development of the behavioral package of the ALABAMA intervention has been published elsewhere[16] . The PAAP targets patients assessed as “low risk” of having a true penicillin allergy, i.e. with no history of anaphylaxis or other severe allergic reactions. It also aimed to streamline the test process by undertaking patient history screening in general practice (Stage 1) and introducing an efficient one-stop procedure at a hospital immunology clinic for PAT. The PAT included either a skin testing (ST) (Stage 2) and oral challenge test (OCT) involving taking oral doses of a penicillin solution while supervised (Stage 3), or just OCT depending on the individual patient

For peer review only - http://bmjopen.bmj.com/site/about/guidelines.xhtml

history. Following the PAT, patients and practices received confirmation of a patient's allergy status by letter from the immunology clinic.

Intervention

Patients in the intervention arm (PAAP arm) received a booklet about allergy testing (Pre-test booklet), completed the two/three stages of PAAP (screening/patient history, skin test and/or oral challenge test) and, if tested negative, received a booklet and a card giving information about their result. The pre-test booklet informed patients about incorrect allergy records, how they may benefit from having a PAT and what was involved. The Post-test booklet informed on the reliability of test results and consequences of a negative test result. A laminated card was provided stating the negative allergy result which could be shown to GPs and other prescribers.

Clinicians' booklet aimed to increase their motivation to refer patients to PAT and prescribe penicillin after a negative PAT result, together with a letter to inform them about the patient test results and how to change allergy records when the test result was negative. A computer pop-up alerting a GP to the change in the patient's allergy status was built into the electronic patient record system (SystmOne, TPP).

Patient and Public Involvement

Alabama programme of work has a Patient Advisory Group advising the research team on all aspects of the study.

Mixed methods evaluation using surveys and interviews

Clinician Survey

Twelve-item surveys on a 7-point Likert scale (Appendix 1) were sent to all clinician participants in the 11 practices. The survey was not validated but its items were informed by constructs from the

For peer review only - http://bmjopen.bmj.com/site/about/guidelines.xhtml

Theoretical Domains Framework (TDF)[17] and measured clinicians' knowledge, cognitive skills, intentions, beliefs about consequences, professional role and identity, goals, and social influences related to PAT. Clinicians were invited to take part in the survey by email at two time points; at the start of the feasibility trial and once all patient participants from a practice had been followed up for 4 months post randomisation. Clinicians completed the survey online (Survey Monkey).

Patient Survey

Surveys were sent to all patient participants in the 11 practices. The patient survey included 13 items on a 7-point Likert scale, where 7 was strongly agree and 1, strongly disagree (Appendix 2). Patients were asked about their beliefs concerning their penicillin allergy status, their views on possible reactions during a PAT, and their intentions to take penicillin in the future in the case of a negative test result.

The aim of the surveys was to compare participants' knowledge and beliefs about penicillin allergy and PAT before and after the feasibility trial. Patients completed the questionnaire at two time points. Patients completed the belief questionnaire at baseline over the telephone with a member of the trial team. They completed the follow up questionnaire 28-30 days post randomisation over the telephone with a member of the trial team.

Analysis

Patient and clinician data were extracted and analysed using SPSS (version 26). Medians for each group and items were reported. A series of non-parametric tests (Wilcoxon Signed Rank Test) were conducted to compare whether the medians for each item when compared to the midpoint of the scale (one sample test), and whether the medians for each item differed between groups (independent samples) and at different time points (related samples).

Interviews

For peer review only

We conducted semi-structured interviews using two topic guides (one for patients, one for clinicians), which were informed by the literature on penicillin allergy[15]. After obtaining consent, interviews were conducted over the telephone by two experienced female qualitative researchers (MW and MS) wo were not part of the trial. The interviews were audio recorded and transcribed verbatim. Interviews continued until data indicated saturation[18].

Clinicians

One clinician from each of the 11 practices was invited to take part in an interview. Clinicians were invited by email. Patients were invited to take part in an interview by letter or telephone. We sampled patients in the intervention arm to include people with positive and negative test results. Clinicians were asked about their views and experience of referring patients to PAT, prescribing penicillin after a negative test result and their review in taking part in the trial and intervention materials.

Patients

Patients were invited to take part in an interview by letter or telephone. We sampled patients in the intervention arm, to include people with positive and negative test results. Patients were asked their views and experiences of undergoing PAT, their experience of taking part in the trial and their views on intervention materials.

Interview analysis

Transcripts were analysed using inductive thematic analysis[19]. Transcripts were read and reread by MW and MS during and after data collection. A coding manual was developed after the first few interviews and was refined during the analytical process. To enhance the trustworthiness of data, analysis was discussed by a multidisciplinary team consisting of psychologists, a primary care

For peer review only - http://bmjopen.bmj.com/site/about/guidelines.xhtml

clinician and colleagues from hospital-based immunology with expertise in penicillin allergy and microbiology services.

Results

Clinician Survey

Fifty-three clinicians responded to the survey (43 GPs, 9 nurse practitioners and 1 pharmacist), response rate of 52.2%. Results for each item are presented in Table 1. Clinicians gave positive responses to the majority of items. In general, they did not have previous experience of referring patients for a PAT, and were unsure whether patients would accept a prescription of penicillin after a negative PAT. Results suggested that GPs and nurses had slightly different beliefs. GPs were more likely than nurses to indicate that they would be happy to refer patients for a PAT, change allergy records and prescribe penicillin, following a negative test result. Nurses seemed less certain that a PAT would benefit their patients, more unsure about the safety of prescribing penicillin after a negative result, more unsure whether colleagues would support de-labelling and less confident about discussing PAT results with patients.

Table 1 Clinician survey responses

	**All respondents**	**GPs (n=43)**	**Nurses (n=9)**	**Pharmacists (n=1)**
Questions	Median (IQR)	Median score (IQR)	Median score(IQR)	Score
1. I understand what is involved in penicillin allergy testing	5 (4 to 6)	5 (4 to 6)	5 (2.5 to 6)	4
2. I have previous experience of referring patients for Penicillin allergy testing	2 (1.5 to 5)	2 (2 to 5)	2 (1.5 to 4)	1
3. I am happy to refer patients for Penicillin allergy testing	6 (5 to 7)	6 (6 to 7)	6 (4 to 7)	2
4. Penicillin allergy testing can benefit my patients	6 (6 to 7)	7 (6 to 7)	6 (5.5 to 7)	6
5. Penicillin allergy testing can benefit my practice	6 (6 to 7)	6 (6 to 7)	6 (6 to 6.5)	6

For peer review only - http://bmjopen.bmj.com/site/about/guidelines.xhtml

6. It is safe to prescribe penicillin after a negative PAT	6 (4 to 6)	6 (5 to 6)	4 (4 to 6.5)	4
7. I am confident in discussing PAT results with my patients	5 (4 to 6)	5 (4 to 6)	4 (4 to 6)	2
8. I am happy to change patient records based on the results of penicillin allergy testing	6 (4.5 to 6)	6 (5 to 7)	5 (4 to 6)	4
9. I would prescribe penicillin, if indicated, to patients with a negative PAT result	6 (5 to 6)	6 (5 to 6)	5 (4 to 6.5)	4
10. My colleagues support de-labelling of patients with incorrect penicillin allergy status using penicillin allergy testing	5 (4 to 6)	6 (4 to 6)	4 (4 to 7)	5
11. My patients would be happy to be referred for penicillin allergy testing	6 (4 to 6)	6 (4 to 6)	5 (4 to 6)	5
12. My patients would be happy to take penicillin following a negative PAT	4 (4 to 6)	4 (4 to 6)	4 (4 to 6)	5

Note: Scale 1= Strongly Disagree, 2= Disagree, 3= Slightly Disagree, 4= Not sure, 5= Slightly Agree, 6= Agree, 7= Strongly agree. PAT, penicillin allergy test

Patient Survey

Seventy-nine patients participated in the feasibility study, of which 36 (46%) completed the baseline questionnaire. Nineteen patients were randomised to the intervention arm. Fifteen of these completed the PAT (2 positive, 13 negative). Ten of these patients completed the follow up survey (all PAT negative). We checked whether survey responses from participants in the intervention and control arms were similar at baseline. As there were no significant differences, we then compared responses at baseline and follow-up for the 10 patients in intervention arm who answered both surveys. Five items indicated that participant beliefs had changed (Table 2).

Patients were already confident about the safety and accuracy of the test at baseline, however they were unsure whether they were severely allergic to penicillin and but did not fear a severe allergic reaction to penicillin.

At follow up patients were more likely to believe that penicillin is the best treatment for bacterial infections; that they would not have a severe allergic reaction to penicillin; and, were less scared of

For peer review only - http://bmjopen.bmj.com/site/about/guidelines.xhtml

having a severe reaction than before they did the test. Moreover, patients were more confident that they were not allergic to penicillin. Lastly, patients were more likely to agree that the majority of patients with penicillin allergy records in the UK might not be allergic.

Table 2 Comparing patient survey responses at baseline and follow-up for patients in the PAAP and usual clinical care arms of the trial.

Questions	Baseline (N=36)			PAAP only (N=10)		
	PAAP Median (N=13) (IQR)	UCC Median (N=23) (IQR)	P value[1]	Baseline (N= 10) Median (IQR)	Follow up (N= 10) Median (IQR)	P value[2]
1. Penicillin is often the best treatment for bacterial infections.	2 (1 to 4)	2 (1 to 4)	.902	2 (1 to 4)	1 (1 to 2)	.026
2. Having an incorrect penicillin allergy label can be harmful for patients.	2 (1 to 2)	2 (1 to 3)	.214	2 (1 to 3)	1 (1 to 2)	.107
3. Having a penicillin allergy test is not beneficial for patients themselves	6 (6 to 7)	6 (5.5 to 6.5)	.931	6 (5.5 to 7)	6 (1.75 to 6.25)	.477
4. Most people in the UK who think they are allergic to penicillin are not actually allergic.	4 (2 to 4)	4 (3 to 4)	-	4 (3.5-6)	2 (1.75 to 4)	.070
5. I believe that I am allergic to penicillin.	4 (4 to 4)	2 (1.5 to 5)	1.000	2 (1.5 to 5)	6 (6 to 6.25)	.012
6. There is a high chance that I would have a serious allergic reaction if I took penicillin.	4 (2 to 4)	4 (3 to 6)	.722	4 (3.5 to 6)	6 (5 to 7)	.031
7. It is important for me to know whether or not I am allergic to penicillin.	1 (1 to 1)	1 (1 to 2)	.610	1 (1 to 1)	1.5 (1 to 2)	.414

For peer review only

For peer review only - http://bmjopen.bmj.com/site/about/guidelines.xhtml

8. Penicillin allergy testing is safe.	2 (2 to 3)	2 (2 to 2)	.368	2 (2 to 2)	1 (1 to 2)	.527
9. Penicillin allergy testing can say if someone is allergic to penicillin or not.	2 (1 to 2)	2 (1.5 to 2)	.756	2 (1.5 to 4)	2 (1 to 2)	.655
10. I would benefit from being able to take penicillin antibiotics safely when needed in future.	1 (1 to 2)	2 (1 to 2)	.951	2 (1 to 2)	1.5 (1 to 2)	-
11. I am frightened about having a serious allergic reaction if I take penicillin.	6 (3 to 6)	5 (3 to 6)	.297	5 (3.5 to 6)	6 (6 to 6.25)	.026
12. My doctor would be happy to prescribe penicillin if a test showed I was not allergic to penicillin	2 (2 to 2)	2 (1.5 to 2)	.464	2 (2 to 2)	2 (1 to 2)	.317
13. I would be happy to take penicillin prescribed by my doctor if a test showed I was not allergic	2 (1 to 2)	2 (1 to 2)	-	2 (1 to 2)	1.5 (1 to 2)	.705

1 = Strongly Agree; 2= Agree; 3= Slightly Agree; 4=not sure; 5= Slightly Disagree; 6= Disagree; 7= Strongly Disagree
1 = Median test, k sample
2= Wilcoxon Signed Rank Test
-= no result

Interviews with patients and clinicians

We conducted 17 interviews, seven interviews with clinicians and 10 with patients between November 2019 and April 2020. Interviews lasted between 15 and 40 minutes. Table 3 presents participant characteristics.

For peer review only - http://bmjopen.bmj.com/site/about/guidelines.xhtml

Table 3 Interview participant characteristics

	Role (clinician only	Mean age (years)	Age range (years)	Gender (Female, n (%))	Role (clinician only	Tested negative (patient only)
Patients (N=10)		63.7	30 to 82	8 (80%)	N/A	8/10
Clinicians (N=7)	6 GPs; 1 Advanced Nurse Practitioner	46.4	43 to 57	4 (57%)	6 GPs; 1 Advanced Nurse Practitioner	N/A

Clinicians

Theme 1: Motivations to join the study: Penicillin allergy is a problem in primary care

Clinicians highlighted that penicillin allergy is a 'major problem' in primary care resulting in fewer antibiotic options for patients and difficulty in prescribing, particularly for patients with multiple allergies and co-morbidities. They were aware that a number of allergy records may be incorrect but had limited access to allergy services. Hence, they saw the study as an opportunity to revoke erroneous labels which otherwise would not be possible.

I think it's a huge problem. […] there's lots of penicillin allergy recorded on patients' notes, a lot of which I think is probably incorrect, but there's no way of proving or disproving it unless they're in this study at the moment or unless this study gets taken further. [P2, GP]

Theme 2: Extent of confidence in allergy testing

Clinicians had confidence in the safety of allergy testing as they believed that the hospital environment was an appropriate place for testing to take place. Also, they reported that testing had components which were familiar to them. Finally, they believed the screening procedures included enough safety netting that patients at risk of a severe reaction would not get to the oral challenge stage:

For peer review only - http://bmjopen.bmj.com/site/about/guidelines.xhtml

Getting to the stage of having to take a penicillin dose orally but there are steps before that to mitigate the risk. I haven't got any particular worries about it, no. [P4, GP]

Theme 3: Experience of discussions with patients about allergy testing

Clinicians felt that the training they received as part of the study allowed them to answer patients' questions. Clinicians found the Pre-test booklet informative and some used it in their discussions with patients, to explain the study and potential benefits from the patients' perspective:

> *Very well prepared actually, from the information provided and also with the information sheets that are given to patients […] I found that very helpful to look up and read beforehand, so the glossy leaflets about what to expect if you were going to the penicillin testing, so it was helpful to read through that so I could talk about that, from a patient's perspective. [P4, GP]*

Clinician's confidence in the safety of testing meant that they also felt comfortable and confident in reassuring patients, if they asked about safety. They also reported that patients who responded to the study were very keen to get tested and find out their allergy status. They highlighted that proactively calling patients to follow-up on the postal invite facilitated recruitment.

Theme 4: Implementation of procedures in a practice: extent of staff involvement

While in each practice there was a GP lead who was the main point of contact for the study team, on a day-to-day basis it meant the champion was often the only person responsible for identifying and consenting patients. GPs highlighted the importance of keeping everyone informed about the study, especially in relation to recording a negative test result and amending allergy records to facilitate future penicillin prescribing:

> *I made sure that all my colleagues knew that any letter regarding the study had to come to me,[…] so I processed all the SystmOne input, made sure that everything was clear. I made a*

For peer review only - http://bmjopen.bmj.com/site/about/guidelines.xhtml

suggestion to the team that I add some additional text regarding the fact that the patient was part of the study and whether they tested positive or not. [P1, GP]

Patients

Theme 1: Reasons for wanting to have access to testing

The main motivation to take part in the study was to have a definite answer on whether they were allergic or not. Patients had varying knowledge about the benefits of penicillin; some were aware that it would be beneficial for them to have access to a wider range of antibiotics and described this as one motivation to get tested. Undergoing testing to help tackle antibiotic resistance was mentioned very rarely and likely prompted by the patient Pre-test booklet. Patients who initially had less knowledge about the benefits of penicillin reported being encouraged to take part after reading the study information:

After reading all the information and hearing that it's one of the best antibiotics, then I just thought, 'Well, if I can actually take it safely, then it's probably a good thing to see if I can get tested or not, so I'll take part.' [P8, Negative allergy test]

Theme 2: Considering the safety of the test

The main concern among patients was the safety of the test. However, they also highlighted that clear and detailed information about testing procedures beforehand was helpful in alleviating their concerns. They valued multiple ways of learning about the process and safety of testing: during the consent procedure with their GP, from trial materials, and from the Pre-test booklet:

[The Pre-test booklet] explained everything that would happen and the time scale and how it would help me and why it would help me; so yes, it affected my decision to take part [P7, Negative allergy test].

For peer review only - http://bmjopen.bmj.com/site/about/guidelines.xhtml

Despite having this information before attending for the test, patients still appreciated having the process explained to them by nurses and clinicians in the hospital. Clear information presented in lay terms put patients at ease.

Theme 3: Acceptance of the test result

Patients seemed to accept the test result, regardless of whether it was positive or negative and indicated that, where applicable, they intended to take penicillin when prescribed in the future. Some explained that they would still exercise caution and look out for any possible signs of allergy while still having confidence in the test:

> *I would sort of think, 'right, I'll take it, I'll make sure that I check everything is okay, you know, am I starting to feel anything.' But because I took it for the few days afterwards and I took quite a few doses, I feel quite okay about it because I didn't get anything at all. So I'd feel quite happy about it. I wouldn't be worried [P4, Negative allergy test]*

Discussion

This study provides an in-depth understanding of patients and primary care clinicians' views of the key barriers and facilitators to referring to and attending PAT and prescribing/taking penicillin after a negative test. It also highlights the positive effects of the behavioural intervention for both patients and clinicians.

Clinicians who took part in the study were motivated by having access to testing as they were convinced of the benefits of penicillin for their patients and their practice. In line with other studies, clinicians were often unsure about criteria for referral or what allergy testing involves[20,21], and reported little prior experience and only some knew what was involved in the test prior to the trial.

Our survey results also suggested that nurses might be more tentative about referring patients and changing an allergy status on medical records. Studies with hospital nurses suggested that they could play a role in antibiotic stewardship activities, including accurate and detailed documentation

For peer review only - http://bmjopen.bmj.com/site/about/guidelines.xhtml

of penicillin allergy[22,23]. However, more studies are needed to explore the views of this group of healthcare professionals and better define the multidisciplinary approach to de-labelling[24] .

While the baseline survey showed that patients had positive beliefs about testing, including safety, these beliefs seemed to be strengthened after they had completed the PAAP. In line with other studies[23-25], qualitative interviews also highlighted the importance of safety of testing as patients valued having numerous opportunities to address their concerns about safety of the test, including conversations with their GP and later with hospital staff.

Our study identified a number of important clinical implications. First, we note the importance of clinician training through provision of educational materials to ensure successful implementation of referral to PAT and subsequent penicillin prescribing in primary care. The information materials provided were useful to clinicians and increased their knowledge and confidence in referring people and having discussions with patients about PAT. Secondly, it also raises the importance of a clear plan in relation to the de-labelling process, involving clear procedures for informing other colleagues about test results to facilitate penicillin prescribing in the future. Our study indicates that communication between staff member is crucial with dedicated roles being key. Thirdly, while patients may have a number of concerns about safety of PAT[13, 21, 26], these concerns can be successfully addressed and educational materials may be useful in increasing patients' motivation; however, primary care clinicians need to be provided with appropriate resources and time to address these issues.

Strengths and limitations

The study benefits from using both survey and interviews, providing complementary perspectives. Including qualitative studies is important in trial and implementation settings, where they are now recommended as part of the mixed methods evaluations[14]

For peer review only - http://bmjopen.bmj.com/site/about/guidelines.xhtml

However, our study would have benefitted from a follow-up survey with clinicians; this was not possible due to study delays. The study recruited from one region in England and includes small numbers due to issues with recruitment and testing availability; hence the results should be extrapolated with caution. Not all trial participants completed the survey, meaning that only views of participants who chose to complete the survey are represented. Both patients and PCP interview participants were a convenience sample recruited within a feasibility study, hence now allowing to capture perspectives of patients who did not want to take part in the interviews, which means that these findings may have to be interpreted cautiously in terms of their transferability to other settings.

Conclusions

This study identified a number and issues of which need to be addressed to ensure successful implementation of penicillin allergy de-labelling programmes. However, the study highlighted that both patients and clinicians' concerns can be addressed with help of carefully designed intervention materials.

Acknowledgments: The ALABAMA team consists of: Jenny Boards, Emily Bongard, Christopher C Butler, Mina Davoudianfar, Mandy East, Joanne Fielding, Philip Howard, Sue H Pavitt, Catherine E Porter, Jonathan AT Sandoe, Marta Santillo, Louise Savic, Sinisa Savic, Bethany Shinkins, Sarah Tonkin-Crine, Marta Wanat, Robert West, and Ly-Mee Yu.

Contributors: Conceptualization, Christopher Butler, Sue Pavitt, Jonathan Sandoe and Sarah Tonkin-Crine; Formal analysis, Marta Wanat, Marta Santillo and Ushma Galal; Funding acquisition, Christopher Butler, Sue Pavitt, Jonathan Sandoe and Sarah Tonkin-Crine; Investigation, Marta Wanat, Marta Santillo, Mina Davoudianfar, Emily Bongard, Sinisa Savic, Louise Savic, Catherine Porter, Joanne Fielding, Christopher Butler, Sue Pavitt, Jonathan Sandoe and Sarah Tonkin-Crine; Methodology, Marta Wanat, Marta Santillo^ and Sarah Tonkin-Crine; Project administration, Catherine Porter and Joanne Fielding; Resources, Sue Pavitt and Jonathan Sandoe; Supervision,

For peer review only - http://bmjopen.bmj.com/site/about/guidelines.xhtml

Sarah Tonkin-Crine; Writing – original draft, Marta Wanat and Marta Santillo; Writing – review & editing, Ushma Galal, Mina Davoudianfar, Emily Bongard, Louise Savic, Catherine Porter, Joanne Fielding, Christopher Butler, Sue Pavitt, Jonathan Sandoe and Sarah Tonkin-Crine.

Funding statement: This study summarises independent research funded by the National Institute for Health Research (NIHR) under its Programme Grants for Applied Research Programme (Grant Reference Number RP-PG-1214-20007). STC received additional funding from the National Institute for Health Research Health Protection Research Unit (NIHR HPRU) in Healthcare Associated Infections and Antimicrobial Resistance at the University of Oxford in partnership with Public Health England (PHE) [HPRU-2012-10041]. The research is supported by the National Institute for Health Research (NIHR) infrastructure at Leeds. The funder had no role in the design of the study; in the collection, analyses, or interpretation of data; in the writing of the manuscript, or in the decision to publish.

Competing interest: None declared. The funders had no role in the design of the study; in the collection, analyses, or interpretation of data; in the writing of the manuscript, or in the decision to publish the results.

Ethics Approval: The study received ethical approval from the London Bridge Research Ethics Committee (Ref: 19/LO/0176). All participants gave informed consent to take part in the study.

Data sharing statement: Additional data are available from authors on reasonable request.

References

1. Shenoy ES, Macy E, Rowe T. et al. Evaluation and management of penicillin allergy: a review. *JAMA* 2019; **321:** 188-99.

2. West R, Smith C, Pavitt S. et al. 'Warning: allergic to penicillin': association between penicillin allergy status in 2.3 million NHS general practice electronic health records, antibiotic prescribing and health outcomes. *J Antimicrob Chemother,* 2019; **74:** 2075-82.

For peer review only - http://bmjopen.bmj.com/site/about/guidelines.xhtml

3. Minh H-BC, Bousquet PJ, Fontaine C, et al. Systemic reactions during skin tests with [beta]-lactams: a risk factor analysis. *J Allergy Clin Immunol* 2006; **117**: 466.

4. Blumenthal KG, Lu N, Zhang Y. et al. Risk of meticillin resistant Staphylococcus aureus and Clostridium difficile in patients with a documented penicillin allergy: population based matched cohort study. *BMJ* 2018; **361**: k2400.

5. Blumenthal KG, Ryan EE, Li Y. et al. The impact of a reported penicillin allergy on surgical site infection risk. *Clin Infect Dis* 2018; **66**: 329-36.

6. Reddy V, Baman NS, Whitener C et al. Drug resistant infections with methicillin-resistant Staphylococcus aureus, Clostridium difficile, and vancomycin resistant Enterococcus are associated with a higher prevalence of penicillin allergy. *J Allergy Clin Immunol* 2013: **131**: AB170.

7. Macy E, & Contreras R. Health care use and serious infection prevalence associated with penicillin "allergy" in hospitalized patients: a cohort study. *J Allergy Clin Immunol* 2014; **133:** 790-96.

8. Charneski L, Deshpande G, Smith et al. Impact of an antimicrobial allergy label in the medical record on clinical outcomes in hospitalized patients. *Pharmacotherapy: J Human Pharm Drug Ther* 2011; **31:** 742-47.

9. Vyles D, Chiu A, Simpson P, Nimmer M, Adams J, Brousseau D. Parent-reported penicillin allergy symptoms in the pediatric emergency department. *Acad Pediatr* 2017; **17:**251-255.

10. Gerace K, Karlin E, McKinnon E, Phillips E. Varying penicillin allergy testing practices in the United States: A time for consensus. *J Allergy Clin Immunol* 2015;**3**: 791-793.

11. Trubiano JA, Beekmann SE, Worth LJ et al. Improving antimicrobial stewardship by antibiotic allergy delabeling: evaluation of knowledge, attitude, and practices throughout the emerging infections network. *Open Forum Infect Dis* 2016; **3:** ofw153.

For peer review only - http://bmjopen.bmj.com/site/about/guidelines.xhtml

12. Gerace K. & Phillips E. Penicillin allergy label persists despite negative testing. *J Allergy Clin Immunol* 2015; **3:** 815-816.

13. Wanat M, Anthierens S, Butler CC et al. Patient and primary care physician perceptions of penicillin allergy testing and subsequent use of penicillin-containing antibiotics: a qualitative study. *J Allergy Clin Immunol Prac* 2019; **7:** 1888-93. e1.

14. Moore G, Audrey S, Barker M, Bond L, Bonell C, Hardeman W, et al. Process evaluation of complex interventions: Medical Research Council guidance. 2014 Presented at: MRC Population Health Science Research Network; 2014; London URL: http://decipher.uk.net/wp-content/uploads/2014/11/MRC-PHSRN-Process-evaluation-guidance.pdf

15. Yardley, L., Morrison, L., Bradbury, K., & Muller, I. (2015). The person-based approach to intervention development: application to digital health-related behavior change interventions. Journal of medical Internet research, **17:** e4055.

16. Santillo M.; Wanat M.; Davoudianfar M. et al. Developing a behavioural intervention package to identify and amend incorrect penicillin allergy records in UK general practice and subsequently change antibiotic use. *BMJ Open* 2020; **10**: e035793. doi: 10.1136/bmjopen-2019-035793.

17. Cane J, O'Connor D, Michie SJIs. Validation of the theoretical domains framework for use in behaviour change and implementation research. *Implement. Sci*. 2012; **7**: 1-17.

18. Fusch PI, Ness LR. Are we there yet? Data saturation in qualitative research. *Qual Rep 2015,* 20: 1408.

19. Braun V, Clarke V. Using thematic analysis in psychology. Qual Res Psychol 2006, **3**: 77-101.

20. Wanat M, Anthierens S, Butler CC.; et al. Patient and prescriber views of penicillin allergy testing and subsequent antibiotic use: a rapid review. Antibiotics 2018; **7**:71.

For peer review only

For peer review only - http://bmjopen.bmj.com/site/about/guidelines.xhtml

21. Hayoun MB, Bourrier T, Pognonec C. et al. Impact du bilan d'allergie aux bêta-lactamines sur les médecins généralistes dans une cohorte de 154 patients. *Revue Française d'Allergologie* 2015; **55**: 333-40.

22. Monsees EA, Tamma PD, Cosgrove SE et al. Integrating bedside nurses into antibiotic stewardship: A practical approach. *Infect Control Hosp Epidemiol* 2019; **40**: 579-84. doi: 10.1017/ice.2018.362 [published Online First: 2019/02/21]

23. Carter, EJ, Greendyke, WG, Furuya, EY et al. Exploring the nurses' role in antibiotic stewardship: a multisite qualitative study of nurses and infection preventionists. Am J Infect Control. 2018; **46:** 492-497.

24. Staicu ML, Vyles D, Shenoy ES et al. Penicillin Allergy Delabeling: A Multidisciplinary Opportunity. *J Allergy Clin Immunol Practice* 2020, **8**:2858-68.e16. doi: https://doi.org/10.1016/j.jaip.2020.04.059

25. Al-Ahmad M, Rodriguez-Bouza TJ. Drug allergy evaluation for betalactam hypersensitivity: Cross-reactivity with cephalosporines, carbapenems and negative predictive value. Asian Pac J *Allergy Immunol* 2018, **36**: 27-31.

26. Phillips EJ, Knowles SR, O'Brien L. et al. The utility of penicillin skin testing in a tertiary care clinic. J *Allergy Clin Immunol* 2002, **109**: S143.

For peer review only

Appendices

Appendix 1 Clinician Penicillin Allergy Belief questionnaire

Please respond to the statements below using the scale provided. Please give only one answer per statement.

I understand what is involved in penicillin allergy testing.

☐	☐	☐	☐	☐	☐	☐
Strongly Agree	Agree	Slightly Agree	Not Sure	Slightly Disagree	Disagree	Strongly Disagree

I have previous experience of referring patients for penicillin allergy testing.

☐	☐	☐	☐	☐	☐	☐
Strongly Agree	Agree	Slightly Agree	Not Sure	Slightly Disagree	Disagree	Strongly Disagree

I am happy to refer patients for penicillin allergy testing.

☐	☐	☐	☐	☐	☐	☐
Strongly Agree	Agree	Slightly Agree	Not Sure	Slightly Disagree	Disagree	Strongly Disagree

Penicillin allergy testing can benefit my patients.

☐	☐	☐	☐	☐	☐	☐
Strongly Agree	Agree	Slightly Agree	Not Sure	Slightly Disagree	Disagree	Strongly Disagree

Penicillin allergy testing can benefit my practice.

☐	☐	☐	☐	☐	☐	☐
Strongly Agree	Agree	Slightly Agree	Not Sure	Slightly Disagree	Disagree	Strongly Disagree

For peer review only

It is safe to prescribe penicillin after a negative penicillin allergy test.

☐	☐	☐	☐	☐	☐	☐
Strongly Agree	Agree	Slightly Agree	Not Sure	Slightly Disagree	Disagree	Strongly Disagree

I am confident in discussing penicillin allergy test results with my patients.

☐	☐	☐	☐	☐	☐	☐
Strongly Agree	Agree	Slightly Agree	Not Sure	Slightly Disagree	Disagree	Strongly Disagree

I am happy to change patient records based on the results of penicillin allergy testing.

☐	☐	☐	☐	☐	☐	☐
Strongly Agree	Agree	Slightly Agree	Not Sure	Slightly Disagree	Disagree	Strongly Disagree

I would prescribe penicillin, if indicated, to patients with a negative penicillin allergy test result.

☐	☐	☐	☐	☐	☐	☐
Strongly Agree	Agree	Slightly Agree	Not Sure	Slightly Disagree	Disagree	Strongly Disagree

My colleagues support de-labelling of patients with incorrect penicillin allergy status using penicillin allergy testing.

☐	☐	☐	☐	☐	☐	☐
Strongly Agree	Agree	Slightly Agree	Not Sure	Slightly Disagree	Disagree	Strongly Disagree

My patients would be happy to be referred for penicillin allergy testing.

☐	☐	☐	☐	☐	☐	☐
Strongly Agree	Agree	Slightly Agree	Not Sure	Slightly Disagree	Disagree	Strongly Disagree

My patients would be happy

For peer review only

to take penicillin following a negative penicillin allergy test.

Strongly Agree	Agree	Slightly Agree	Not Sure	Slightly Disagree	Disagree	Strongly Disagree
☐	☐	☐	☐	☐	☐	☐

Appendix 2 Patient Penicillin Allergy Belief questionnaire

Below statements to be asked to patients with responses given as Likert scale (1-7):

14. Penicillin is often the best treatment for bacterial infections.

Strongly Agree	Agree	Slightly Agree	Not Sure	Slightly Disagree	Disagree	Strongly Disagree
☐	☐	☐	☐	☐	☐	☐

15. Having an incorrect penicillin allergy label can be harmful for patients.

Strongly Agree	Agree	Slightly Agree	Not Sure	Slightly Disagree	Disagree	Strongly Disagree
☐	☐	☐	☐	☐	☐	☐

16. Having a penicillin allergy test is not beneficial for patients themselves.

Strongly Agree	Agree	Slightly Agree	Not Sure	Slightly Disagree	Disagree	Strongly Disagree
☐	☐	☐	☐	☐	☐	☐

17. Most people in the UK who think they are allergic to penicillin are not actually allergic.

Strongly Agree	Agree	Slightly Agree	Not Sure	Slightly Disagree	Disagree	Strongly Disagree
☐	☐	☐	☐	☐	☐	☐

For peer review only

18. I believe that I am allergic to penicillin.

☐	☐	☐	☐	☐	☐	☐
Strongly Agree	Agree	Slightly Agree	Not Sure	Slightly Disagree	Disagree	Strongly Disagree

19. There is a high chance that I would have a serious allergic reaction if I took penicillin.

☐	☐	☐	☐	☐	☐	☐
Strongly Agree	Agree	Slightly Agree	Not Sure	Slightly Disagree	Disagree	Strongly Disagree

20. It is important for me to know whether or not I am allergic to penicillin.

☐	☐	☐	☐	☐	☐	☐
Strongly Agree	Agree	Slightly Agree	Not Sure	Slightly Disagree	Disagree	Strongly Disagree

21. Penicillin allergy testing is safe.

☐	☐	☐	☐	☐	☐	☐
Strongly Agree	Agree	Slightly Agree	Not Sure	Slightly Disagree	Disagree	Strongly Disagree

22. Penicillin allergy testing can say if someone is allergic to penicillin or not.

☐	☐	☐	☐	☐	☐	☐
Strongly Agree	Agree	Slightly Agree	Not Sure	Slightly Disagree	Disagree	Strongly Disagree

23. I would benefit from being able to take penicillin antibiotics safely when needed in future.

☐	☐	☐	☐	☐	☐	☐
Strongly Agree	Agree	Slightly Agree	Not Sure	Slightly Disagree	Disagree	Strongly Disagree

24. I am frightened about having a serious allergic reaction if I take penicillin.

☐	☐	☐	☐	☐	☐	☐
Strongly Agree	Agree	Slightly Agree	Not Sure	Slightly Disagree	Disagree	Strongly Disagree

For peer review only

25. My doctor would be happy to prescribe penicillin if a test showed I was not allergic to penicillin.

Strongly Agree	Agree	Slightly Agree	Not Sure	Slightly Disagree	Disagree	Strongly Disagree
☐	☐	☐	☐	☐	☐	☐

26. I would be happy to take penicillin prescribed by my doctor if a test showed I was not allergic.

Strongly Agree	Agree	Slightly Agree	Not Sure	Slightly Disagree	Disagree	Strongly Disagree
☐	☐	☐	☐	☐	☐	☐

For peer review only

COREQ (COnsolidated criteria for REporting Qualitative research) Checklist

A checklist of items that should be included in reports of qualitative research. You must report the page number in your manuscript where you consider each of the items listed in this checklist. If you have not included this information, either revise your manuscript accordingly before submitting or note N/A.

Topic	Item No.	Guide Questions/Description	Reported on Page No.
**Domain 1: Research team and reflexivity**			
Personal characteristics			
Interviewer/facilitator	1	Which author/s conducted the interview or focus group?	Page 6
Credentials	2	What were the researcher's credentials? E.g. PhD, MD	page 6
Occupation	3	What was their occupation at the time of the study?	page 6
Gender	4	Was the researcher male or female?	page 6
Experience and training	5	What experience or training did the researcher have?	page 6
Relationship with participants			
Relationship established	6	Was a relationship established prior to study commencement?	page 6
Participant knowledge of the interviewer	7	What did the participants know about the researcher? e.g. personal goals, reasons for doing the research	page 6
Interviewer characteristics	8	What characteristics were reported about the inter viewer/facilitator? e.g. Bias, assumptions, reasons and interests in the research topic	page 6
**Domain 2: Study design**			
Theoretical framework			
Methodological orientation and Theory	9	What methodological orientation was stated to underpin the study? e.g. grounded theory, discourse analysis, ethnography, phenomenology, content analysis	page 7
Participant selection			
Sampling	10	How were participants selected? e.g. purposive, convenience, consecutive, snowball	page 7
Method of approach	11	How were participants approached? e.g. face-to-face, telephone, mail, email	page 7
Sample size	12	How many participants were in the study?	page 11
Non-participation	13	How many people refused to participate or dropped out? Reasons?	page 11
Setting			
Setting of data collection	14	Where was the data collected? e.g. home, clinic, workplace	page 6
Presence of non-participants	15	Was anyone else present besides the participants and researchers?	N/A
Description of sample	16	What are the important characteristics of the sample? e.g. demographic data, date	page 11
Data collection			
Interview guide	17	Were questions, prompts, guides provided by the authors? Was it pilot tested?	Appendix
Repeat interviews	18	Were repeat inter views carried out? If yes, how many?	n/a
Audio/visual recording	19	Did the research use audio or visual recording to collect the data?	Page 6
Field notes	20	Were field notes made during and/or after the inter view or focus group?	Page 7
Duration	21	What was the duration of the inter views or focus group?	Page 11
Data saturation	22	Was data saturation discussed?	Page 6
Transcripts returned	23	Were transcripts returned to participants for comment and/or	No

For peer review only

Topic	Item No.	Guide Questions/Description	Reported on Page No.
		correction?	
**Domain 3: analysis and findings**			
Data analysis			
Number of data coders	24	How many data coders coded the data?	page 7
Description of the coding tree	25	Did authors provide a description of the coding tree?	pages 12-14
Derivation of themes	26	Were themes identified in advance or derived from the data?	page 7
Software	27	What software, if applicable, was used to manage the data?	n/a
Participant checking	28	Did participants provide feedback on the findings?	n/a
Reporting			
Quotations presented	29	Were participant quotations presented to illustrate the themes/findings? Was each quotation identified? e.g. participant number	pages 12-14
Data and findings consistent	30	Was there consistency between the data presented and the findings?	pages 12-14
Clarity of major themes	31	Were major themes clearly presented in the findings?	pages 12-14
Clarity of minor themes	32	Is there a description of diverse cases or discussion of minor themes?	pages 12-14

Developed from: Tong A, Sainsbury P, Craig J. Consolidated criteria for reporting qualitative research (COREQ): a 32-item checklist for interviews and focus groups. *International Journal for Quality in Health Care*. 2007. Volume 19, Number 6: pp. 349 – 357

Once you have completed this checklist, please save a copy and upload it as part of your submission. DO NOT include this checklist as part of the main manuscript document. It must be uploaded as a separate file.

For peer review only

BMJ Open

Mixed-methods evaluation of a behavioural intervention package to identify and amend incorrect penicillin allergy records in UK general practice

Journal:	*BMJ Open*
Manuscript ID	bmjopen-2021-057471.R1
Article Type:	Original research
Date Submitted by the Author:	18-Mar-2022
Complete List of Authors:	Wanat, Marta; Oxford University, Nuffield Department of Primary Care Health Sciences<br>Santillo, Marta; Oxford University, Nuffield Department of Primary Care Health Sciences<br>Galal, Ushma; University of Oxford, Nuffield Department of Primary Care Health Sciences<br>Davoudianfar, Mina; University of Oxford, Nuffield Department of Primary Care Health Sciences<br>Bongard, Emily; University of Oxford, Nuffield Department of Primary Care Health Sciences<br>Savic, Sinisa; University of Leeds Faculty of Medicine and Health,<br>Savic, Louise; University of Leeds Faculty of Medicine and Health<br>Porter, Catherine; University of Leeds, Healthcare Associated Infection Group, University of Leeds and Leeds Teaching Hospitals NHS Trust, Leeds LS1 3EX, UK<br>Fielding, Joanne; University of Leeds, Healthcare Associated Infection Group, University of Leeds and Leeds Teaching Hospitals NHS Trust, Leeds LS1 3EX, UK<br>Butler, Christopher C.; University of Oxford, Nuffield Department of Primary Health Care Sciences<br>Pavitt, Sue; University of Leeds, Dental Translational and Clinical Research Unit, Faculty of Medicine and Health,<br>Sandoe, Jonathan; University of Leeds Faculty of Medicine and Health, Healthcare Associated Infection Group, University of Leeds and Leeds Teaching Hospitals NHS Trust, Leeds LS1 3EX, UK<br>Tonkin-Crine, Sarah ; University of Oxford, Nuffield Department of Primary Care Health Science; National Institute for Health Research (NIHR) Health Protection Research Unit (HPRU) in Healthcare Associated Infections and Antimicrobial Resistance, University of Oxford, Oxford, UK
**Primary Subject Heading**:	Immunology (including allergy)
Secondary Subject Heading:	General practice / Family practice, Immunology (including allergy)
Keywords:	PRIMARY CARE, IMMUNOLOGY, QUALITATIVE RESEARCH

SCHOLARONE™
Manuscripts

I, the Submitting Author has the right to grant and does grant on behalf of all authors of the Work (as defined in the below author licence), an exclusive licence and/or a non-exclusive licence for contributions from authors who are: i) UK Crown employees; ii) where BMJ has agreed a CC-BY licence shall apply, and/or iii) in accordance with the terms applicable for US Federal Government officers or employees acting as part of their official duties; on a worldwide, perpetual, irrevocable, royalty-free basis to BMJ Publishing Group Ltd ("BMJ") its licensees and where the relevant Journal is co-owned by BMJ to the co-owners of the Journal, to publish the Work in this journal and any other BMJ products and to exploit all rights, as set out in our licence.

The Submitting Author accepts and understands that any supply made under these terms is made by BMJ to the Submitting Author unless you are acting as an employee on behalf of your employer or a postgraduate student of an affiliated institution which is paying any applicable article publishing charge ("APC") for Open Access articles. Where the Submitting Author wishes to make the Work available on an Open Access basis (and intends to pay the relevant APC), the terms of reuse of such Open Access shall be governed by a Creative Commons licence – details of these licences and which Creative Commons licence will apply to this Work are set out in our licence referred to above.

Other than as permitted in any relevant BMJ Author's Self Archiving Policies, I confirm this Work has not been accepted for publication elsewhere, is not being considered for publication elsewhere and does not duplicate material already published. I confirm all authors consent to publication of this Work and authorise the granting of this licence.

For peer review only

Mixed-methods evaluation of a behavioural intervention package to identify and amend incorrect penicillin allergy records in UK general practice

Wanat, M.* [1], Santillo, M.* [1], Galal, U. [1], Davoudianfar, M. [1], Bongard, E. [1], Savic, S. [2], Savic, L. [2], Porter, C. E. [3], Fielding, J. [3], Butler C. [1], Pavitt, S. H. [4], Sandoe, J. A. T. [3$], Tonkin-Crine, S. [1,5$] On behalf of the ALABAMA team.

*Joint first authors

$Joint senior authors

Correspondence to: Dr Marta Wanat, Nuffield Department of Primary Care Health Sciences, University of Oxford, Radcliffe Observatory Quarter, Woodstock Road, Oxford, OX2 6GG , UK; email: marta.wanat@phc.ox.ac.uk

[1] Nuffield Department of Primary Care Health Sciences, University of Oxford, Radcliffe Observatory Quarter, Woodstock Road, Oxford, OX2 6GG , UK

[2] Faculty of Medicine and Health, University of Leeds and Leeds Teaching Hospitals NHS Trust, Leeds LS1 3EX, UK

[3] Healthcare Associated Infection Group, University of Leeds and Leeds Teaching Hospitals NHS Trust, Leeds LS1 3EX, UK

[4] Dental Translational and Clinical Research Unit, Faculty of Medicine and Health, Worsley Building, Clarendon Way, University of Leeds, LS2 9LU, UK

[5] NIHR Health Protection Research Unit in Healthcare Associated Infections and Antimicrobial Resistance, University of Oxford, UK

For peer review only

For peer review only - http://bmjopen.bmj.com/site/about/guidelines.xhtml

Abstract

Objectives About 6% of the UK general practice population has a record of a penicillin allergy but fewer than 10% of these are likely to be truly allergic. In the ALABAMA (Allergy Antibiotics and Microbial resistance) feasibility trial, randomised primary care patients with penicillin allergy were randomised to penicillin allergy assessment pathway (PAAP) or usual care to assess the effect on health outcomes. A behavioural intervention package was developed to aid de-labelling. This study aimed to investigate patients' and clinicians' views of Penicillin Allergy Testing (PAT).

Design We conducted a mixed-methods process evaluation embedded within the ALABAMA trial which included a clinician survey, a patient survey (at baseline and follow up) and semi-structured interviews with patients and clinicians.

Settings The study was conducted in primary care, as part of the feasibility stage of the ALABAMA trial.

Participants Patients and primary care clinicians.

Results Clinicians (N=53; 52.2%) were positive about PAT and its potential value but did not have previous experience of referring patients for a PAT and were unsure whether patients would take penicillin after a negative allergy test. Patients (N=36; 46%) were unsure whether they were severely allergic to penicillin and did not fear a severe allergic reaction to penicillin. Clinician interviews showed that they were already aware of the benefit of PAT. Interviews with patients suggested the importance of safety as patients valued having numerous opportunities to address their concerns about safety of the test.

Conclusions This study highlights the positive effects of the ALABAMA behavioural intervention for both patients and clinicians.

Keywords: primary care, immunology, qualitative research

For peer review only - http://bmjopen.bmj.com/site/about/guidelines.xhtml

Strengths and limitations of this study

- The study benefits from using both survey and interviews, providing complementary perspectives.
- The response rate for both clinician (52.2%) and patient surveys (46%) was relatively low.
- The study would have benefitted from a follow-up survey with clinicians, which was not possible due to study delays.
- The study recruited from one region in England and includes small numbers due to issues with recruitment and testing availability; hence the results should be extrapolated with caution.
- Not all trial participants completed the survey, meaning that only views of participants who chose to complete the survey are represented; additionally, both patient and clinician interview participants were a convenience sample recruited within a feasibility study, hence not allowing to capture perspectives of individuals who did not want to take part in the interviews.

Introduction

Six to 10% of primary care patients in the UK have a penicillin allergy record[1,2] but fewer than 10% of these patients are likely to be allergic when formally tested,[3] which means that a large proportion of these people are unnecessarily avoiding first-line antibiotic treatments. Penicillin allergy testing (PAT), usually undertaken in specialist immunology clinics, offers the opportunity to confirm or discount a penicillin allergy, although such services are only currently available for a limited number of patients. The negative consequences of having a penicillin allergy record include: suboptimal clinical outcomes, increased antibiotic resistance[4-6] and higher healthcare costs.[5,7,8]

For peer review only

For peer review only - http://bmjopen.bmj.com/site/about/guidelines.xhtml

Barriers to removing incorrect penicillin allergy records include lack of standardised protocols across practices,[9, 10] lack of access to skin testing,[11] and the difficulty of convincing patients that the record is no longer needed after a negative test.[12,13] These studies also suggest multiple ways to improve research, testing and implementation of penicillin allergy de-labelling initiatives such as easier access to skin testing reagents, standardised skin testing protocols and a greater understanding of penicillin allergy epidemiology.

There is limited understanding of the views of patients and primary care healthcare professionals on de-labelling initiatives in primary care. Specifically, it would be helpful to have a better understanding of their views about simplifying and standardizing skin testing protocols for use only in high risk patients and performing routine direct oral challenges in individuals with low-risk histories to confirm current tolerance. It is necessary to develop a greater appreciation in patients and clinicians of the well-established epidemiology of suspected penicillin allergy, particularly how rarely it is confirmed. The NIHR-funded ALABAMA programme includes a randomised controlled trial of primary care patients who are randomised to a pre-emptive Penicillin Allergy Assessment pathway (PAAP) or usual clinical care to assess the effect on health outcomes.

Within this study, we addressed the current gap, by investigating the perspective of both patients and clinicians on penicillin allergy testing as part of the mixed-methods process evaluation [14,15] of the ALABAMA feasibility trial. Our mixed-methods process evaluation included a clinician questionnaire, a patient questionnaire, and interviews with clinicians and patients.

Methods

The ALABAMA feasibility trial involved 11 general practices based in the Leeds/Bradford area of England. The behavioural intervention package was designed to target both general practice clinicians responsible for the prescription of antibiotics and patients with a suspected incorrect penicillin allergy record, and was an integral part of the PAAP. The details on the process, methods and outcomes of the development of the behavioral package of the ALABAMA intervention has been

For peer review only - http://bmjopen.bmj.com/site/about/guidelines.xhtml

published elsewhere[16]. The PAAP targets patients assessed as “low risk” of having a true penicillin allergy, i.e. with no history of anaphylaxis or other severe allergic reactions. It also aimed to streamline the test process by undertaking patient history screening in general practice (Stage 1) and introducing an efficient one-stop procedure at a hospital immunology clinic for PAT. The PAT included either a skin testing (ST) (Stage 2) and oral challenge test (OCT) involving taking oral doses of a penicillin solution while supervised (Stage 3), or-proceeding directly to an OCT , depending on the individual patient history (the majority of patients). Following the PAT, patients and practices received confirmation of a patient’s allergy status by letter from the immunology clinic.

Intervention

Patients in the intervention arm (PAAP arm) received a booklet about allergy testing (Pre-test booklet), completed the two/three stages of PAAP (screening/patient history, skin test and/or oral challenge test) and, if tested negative, received a booklet and a card giving information about their result. The pre-test booklet informed patients about incorrect allergy records, how they may benefit from having a PAT and what was involved. The Post-test booklet informed on the reliability of test results and consequences of a negative test result. A laminated card was provided stating the negative allergy result which could be shown to GPs and other prescribers.

Clinicians’ booklet aimed to increase their motivation to refer patients to PAT and prescribe penicillin after a negative PAT result, together with a letter to inform them about the patient test results and how to change allergy records when the test result was negative. A computer pop-up alerting a GP to the change in the patient’s allergy status was built into the electronic patient record system (SystmOne, TPP).

Patient and public involvement

The ALABAMA programme of work has a Patient Advisory Group advising the research team on all aspects of the study.

For peer review only - http://bmjopen.bmj.com/site/about/guidelines.xhtml

Mixed methods evaluation using surveys and interviews

Clinician survey

Twelve-item surveys on a 7-point Likert scale (Appendix 1) were sent to all clinician participants in the 11 practices. The survey was not validated but its items were informed by constructs from the Theoretical Domains Framework (TDF)[17] and measured clinicians' knowledge, cognitive skills, intentions, beliefs about consequences, professional role and identity, goals, and social influences related to PAT. Clinicians were invited to take part in the survey by email at two time points; at the start of the feasibility trial and once all patient participants from a practice had been followed up for 4 months post randomisation. Clinicians completed the survey online (Survey Monkey).

Patient survey

Surveys were sent to all patient participants in the 11 practices. The patient survey included 13 items on a 7-point Likert scale, where 7 was strongly agree and 1, strongly disagree (Appendix 2). Patients were asked about their beliefs concerning their penicillin allergy status, their views on possible reactions during a PAT, and their intentions to take penicillin in the future in the case of a negative test result.

The aim of the surveys was to compare participants' knowledge and beliefs about penicillin allergy and PAT before and after the feasibility trial. Patients completed the questionnaire at two time points. Patients completed the belief questionnaire at baseline over the telephone with a member of the trial team. They completed the follow up questionnaire 28-30 days post randomisation over the telephone with a member of the trial team.

Analysis

Patient and clinician data were extracted and analysed using SPSS (version 26). Medians for each group and items were reported. A series of non-parametric tests (Wilcoxon Signed Rank Test) were

For peer review only - http://bmjopen.bmj.com/site/about/guidelines.xhtml

conducted to compare whether the medians for each item when compared to the midpoint of the scale (one sample test), and whether the medians for each item differed between groups (independent samples) and at different time points (related samples).

Interviews

We conducted semi-structured interviews using two topic guides (one for patients, one for clinicians; Appendix 3), which were informed by the literature on penicillin allergy[15]. After obtaining written consent, interviews were conducted over the telephone by two experienced female qualitative researchers (MW and MS) wo were not part of the trial. The interviews were audio recorded and transcribed verbatim. Interviews continued until data indicated saturation[18].

Clinicians

One clinician from each of the 11 practices was invited to take part in an interview. This was the study champion, who led on the patient recruitment and was often responsible for updating patients' records. Clinicians were invited by email. Clinicians were asked about their views and experience of referring patients to PAT, prescribing penicillin after a negative test result and their experience in taking part in the trial and intervention materials.

Patients

Patients were invited to take part in an interview by letter or telephone. We sampled patients in the intervention arm, to include people with positive and negative test results. Patients were asked their views and experiences of undergoing PAT, their experience of taking part in the trial and their views on intervention materials.

Interview analysis

For peer review only - http://bmjopen.bmj.com/site/about/guidelines.xhtml

Transcripts were analysed using inductive thematic analysis[19]. Transcripts were read and reread by MW and MS during and after data collection. A coding manual was developed after the first few interviews and was refined during the analytical process. To enhance the trustworthiness of data, analysis was discussed by a multidisciplinary team consisting of psychologists, a primary care clinician and colleagues from hospital-based immunology with expertise in penicillin allergy and microbiology services.

Results

Clinician survey

Fifty-three clinicians responded to the survey (43 GPs, 9 nurse practitioners and 1 pharmacist), response rate of 52.2%. Results for each item are presented in Table 1. Clinicians gave positive responses to the majority of items. In general, they did not have previous experience of referring patients for a PAT, and were unsure whether patients would accept a prescription of penicillin after a negative PAT. Results suggested that GPs and nurses had slightly different beliefs. GPs were more likely than nurses to indicate that they would be happy to refer patients for a PAT, change allergy records and prescribe penicillin, following a negative test result. Nurses seemed less certain that a PAT would benefit their patients, more unsure about the safety of prescribing penicillin after a negative result, more unsure whether colleagues would support de-labelling and less confident about discussing PAT results with patients.

Table 1. Clinician survey responses

	All respondents	GPs (n=43)	Nurses (n=9)	Pharmacists (n=1)
Questions	Median (IQR)	Median score (IQR)	Median score(IQR)	Score
1. I understand what is involved in penicillin allergy testing	5 (4 to 6)	5 (4 to 6)	5 (2.5 to 6)	4

For peer review only - http://bmjopen.bmj.com/site/about/guidelines.xhtml

2. I have previous experience of referring patients for Penicillin allergy testing	2 (1.5 to 5)	2 (2 to 5)	2 (1.5 to 4)	1
3. I am happy to refer patients for Penicillin allergy testing	6 (5 to 7)	6 (6 to 7)	6 (4 to 7)	2
4. Penicillin allergy testing can benefit my patients	6 (6 to 7)	7 (6 to 7)	6 (5.5 to 7)	6
5. Penicillin allergy testing can benefit my practice	6 (6 to 7)	6 (6 to 7)	6 (6 to 6.5)	6
6. It is safe to prescribe penicillin after a negative PAT	6 (4 to 6)	6 (5 to 6)	4 (4 to 6.5)	4
7. I am confident in discussing PAT results with my patients	5 (4 to 6)	5 (4 to 6)	4 (4 to 6)	2
8. I am happy to change patient records based on the results of penicillin allergy testing	6 (4.5 to 6)	6 (5 to 7)	5 (4 to 6)	4
9. I would prescribe penicillin, if indicated, to patients with a negative PAT result	6 (5 to 6)	6 (5 to 6)	5 (4 to 6.5)	4
10. My colleagues support de-labelling of patients with incorrect penicillin allergy status using penicillin allergy testing	5 (4 to 6)	6 (4 to 6)	4 (4 to 7)	5
11. My patients would be happy to be referred for penicillin allergy testing	6 (4 to 6)	6 (4 to 6)	5 (4 to 6)	5
12. My patients would be happy to take penicillin following a negative PAT	4 (4 to 6)	4 (4 to 6)	4 (4 to 6)	5

Note: Scale 1= Strongly Disagree, 2= Disagree, 3= Slightly Disagree, 4= Not sure, 5= Slightly Agree, 6= Agree, 7= Strongly agree. PAT, penicillin allergy test

Patient survey

Seventy-nine patients participated in the feasibility study, of which 36 (46%) completed the baseline questionnaire. Nineteen patients were randomised to the intervention arm. Fifteen of these completed the PAT (2 positive, 13 negative). Ten of these patients completed the follow up survey (all PAT negative). We checked whether survey responses from participants in the intervention and control arms were similar at baseline. As there were no significant differences, we then compared responses at baseline and follow-up for the 10 patients in intervention arm who answered both surveys. Five items indicated that participant beliefs had changed (Table 2).

For peer review only

For peer review only - http://bmjopen.bmj.com/site/about/guidelines.xhtml

Patients were already confident about the safety and accuracy of the test at baseline, however they were unsure whether they were severely allergic to penicillin and ~~but~~ did not fear a severe allergic reaction to penicillin.

At follow up patients were more likely to believe that penicillin is the best treatment for bacterial infections; that they would not have a severe allergic reaction to penicillin; and, were less scared of having a severe reaction than before they did the test. Moreover, patients were more confident that they were not allergic to penicillin. Lastly, patients were more likely to agree that the majority of patients with penicillin allergy records in the UK might not be allergic.

Table 2. Comparing patient survey responses at baseline and follow-up for patients in the PAAP and usual clinical care arms of the trial

Questions	Baseline (N=36)			PAAP only (N=10)		
	PAAP Median (N=13) (IQR)	UCC Median (N=23) (IQR)	P value[1]	Baseline (N= 10) Median (IQR)	Follow up (N= 10) Median (IQR)	P value[2]
1. Penicillin is often the best treatment for bacterial infections.	2 (1 to 4)	2 (1 to 4)	.902	2 (1 to 4)	1 (1 to 2)	.026
2. Having an incorrect penicillin allergy label can be harmful for patients.	2 (1 to 2)	2 (1 to 3)	.214	2 (1 to 3)	1 (1 to 2)	.107
3. Having a penicillin allergy test is not beneficial for patients themselves	6 (6 to 7)	6 (5.5 to 6.5)	.931	6 (5.5 to 7)	6 (1.75 to 6.25)	.477
4. Most people in the UK who think they are allergic to penicillin are not actually allergic.	4 (2 to 4)	4 (3 to 4)	-	4 (3.5-6)	2 (1.75 to 4)	.070

For peer review only - http://bmjopen.bmj.com/site/about/guidelines.xhtml

5. I believe that I am allergic to penicillin.	4 (4 to 4)	2 (1.5 to 5)	1.000	2 (1.5 to 5)	6 (6 to 6.25)	.012
6. There is a high chance that I would have a serious allergic reaction if I took penicillin.	4 (2 to 4)	4 (3 to 6)	.722	4 (3.5 to 6)	6 (5 to 7)	.031
7. It is important for me to know whether or not I am allergic to penicillin.	1 (1 to 1)	1 (1 to 2)	.610	1 (1 to 1)	1.5 (1 to 2)	.414
8. Penicillin allergy testing is safe.	2 (2 to 3)	2 (2 to 2)	.368	2 (2 to 2)	1 (1 to 2)	.527
9. Penicillin allergy testing can say if someone is allergic to penicillin or not.	2 (1 to 2)	2 (1.5 to 2)	.756	2 (1.5 to 4)	2 (1 to 2)	.655
10. I would benefit from being able to take penicillin antibiotics safely when needed in future.	1 (1 to 2)	2 (1 to 2)	.951	2 (1 to 2)	1.5 (1 to 2)	-
11. I am frightened about having a serious allergic reaction if I take penicillin.	6 (3 to 6)	5 (3 to 6)	.297	5 (3.5 to 6)	6 (6 to 6.25)	.026
12. My doctor would be happy to prescribe penicillin if a test showed I was not allergic to penicillin	2 (2 to 2)	2 (1.5 to 2)	.464	2 (2 to 2)	2 (1 to 2)	.317
13. I would be happy to take penicillin prescribed by my doctor if a test showed I was not allergic	2 (1 to 2)	2 (1 to 2)	-	2 (1 to 2)	1.5 (1 to 2)	.705

1 = Strongly Agree; 2= Agree; 3= Slightly Agree; 4=not sure; 5= Slightly Disagree; 6= Disagree; 7= Strongly Disagree

1 = Median test, k sample

2= Wilcoxon Signed Rank Test

-= no result

Interviews with patients and clinicians

For peer review only - http://bmjopen.bmj.com/site/about/guidelines.xhtml

We conducted 17 interviews, seven interviews with clinicians and 10 with patients. We invited 12 patients to an interview but two have not responded to an invitation. The interviews have been conducted between November 2019 and April 2020 and lasted between 15 and 40 minutes. Table 3 presents participant characteristics.

Table 3. Interview participant characteristics

	Role (clinician only	Mean age (years)	Age range (years)	Gender (Female, n (%))	Role (clinician only	Tested negative (patient only)
Patients (N=10)		63.7	30 to 82	8 (80%)	N/A	8/10
Clinicians (N=7)	6 GPs; 1 Advanced Nurse Practitioner	46.4	43 to 57	4 (57%)	6 GPs; 1 Advanced Nurse Practitioner	N/A

Clinicians

<u>*Theme 1: Motivations to join the study: Penicillin allergy is a problem in primary care*</u>

Clinicians highlighted that penicillin allergy is a 'major problem' in primary care resulting in fewer antibiotic options for patients and difficulty in prescribing, particularly for patients with multiple allergies and co-morbidities. They were aware that a number of allergy records may be incorrect but had limited access to allergy services. Hence, they saw the study as an opportunity to revoke erroneous labels which otherwise would not be possible.

I think it's a huge problem. […] there's lots of penicillin allergy recorded on patients' notes, a lot of which I think is probably incorrect, but there's no way of proving or disproving it unless they're in this study at the moment or unless this study gets taken further. [P2, GP]

<u>*Theme 2: Extent of confidence in allergy testing*</u>

Clinicians had confidence in the safety of allergy testing as they believed that the hospital environment was an appropriate place for testing to take place. Also, they reported that testing had

For peer review only - http://bmjopen.bmj.com/site/about/guidelines.xhtml

components which were familiar to them. Finally, they believed the screening procedures included enough safety netting that patients at risk of a severe reaction would not get to the oral challenge stage:

Getting to the stage of having to take a penicillin dose orally but there are steps before that to mitigate the risk. I haven't got any particular worries about it, no. [P4, GP]

Theme 3: Experience of discussions with patients about allergy testing

Clinicians felt that the training they received as part of the study allowed them to answer patients' questions. Clinicians found the Pre-test booklet informative and some used it in their discussions with patients, to explain the study and potential benefits from the patients' perspective:

> *Very well prepared actually, from the information provided and also with the information sheets that are given to patients [...] I found that very helpful to look up and read beforehand, so the glossy leaflets about what to expect if you were going to the penicillin testing, so it was helpful to read through that so I could talk about that, from a patient's perspective. [P4, GP]*

Clinician's confidence in the safety of testing meant that they also felt comfortable and confident in reassuring patients, if they asked about safety. They also reported that patients who responded to the study were very keen to get tested and find out their allergy status. They highlighted that proactively calling patients to follow-up on the postal invite facilitated recruitment.

Theme 4: Implementation of procedures in a practice: extent of staff involvement

While in each practice there was a GP lead who was the main point of contact for the study team, on a day-to-day basis it meant the champion was often the only person responsible for identifying and consenting patients. GPs highlighted the importance of keeping everyone informed about the study, especially in relation to recording a negative test result and amending allergy records to facilitate future penicillin prescribing:

For peer review only - http://bmjopen.bmj.com/site/about/guidelines.xhtml

I made sure that all my colleagues knew that any letter regarding the study had to come to me,[…] so I processed all the SystmOne input, made sure that everything was clear. I made a suggestion to the team that I add some additional text regarding the fact that the patient was part of the study and whether they tested positive or not. [P1, GP]

Patients

<u>*Theme 1: Reasons for wanting to have access to testing*</u>

The main motivation to take part in the study was to have a definite answer on whether they were allergic or not. Patients had varying knowledge about the benefits of penicillin; some were aware that it would be beneficial for them to have access to a wider range of antibiotics and described this as one motivation to get tested. Undergoing testing to help tackle antibiotic resistance was mentioned very rarely and likely prompted by the patient Pre-test booklet. Patients who initially had less knowledge about the benefits of penicillin reported being encouraged to take part after reading the study information:

After reading all the information and hearing that it's one of the best antibiotics, then I just thought, 'Well, if I can actually take it safely, then it's probably a good thing to see if I can get tested or not, so I'll take part.' [P8, Negative allergy test]

<u>*Theme 2: Considering the safety of the test*</u>

The main concern among patients was the safety of the test. However, they also highlighted that clear and detailed information about testing procedures beforehand was helpful in alleviating their concerns. They valued multiple ways of learning about the process and safety of testing: during the consent procedure with their GP, from trial materials, and from the Pre-test booklet:

[The Pre-test booklet] explained everything that would happen and the time scale and how it would help me and why it would help me; so yes, it affected my decision to take part [P7, Negative allergy test].

For peer review only - http://bmjopen.bmj.com/site/about/guidelines.xhtml

Despite having this information before attending for the test, patients still appreciated having the process explained to them by nurses and clinicians in the hospital. Clear information presented in lay terms put patients at ease.

Theme 3: Acceptance of the test result

Patients seemed to accept the test result, regardless of whether it was positive or negative and indicated that, where applicable, they intended to take penicillin when prescribed in the future. Some explained that they would still exercise caution and look out for any possible signs of allergy while still having confidence in the test:

> *I would sort of think, 'right, I'll take it, I'll make sure that I check everything is okay, you know, am I starting to feel anything.' But because I took it for the few days afterwards and I took quite a few doses, I feel quite okay about it because I didn't get anything at all. So I'd feel quite happy about it. I wouldn't be worried [P4, Negative allergy test]*

Discussion

This study provides an in-depth understanding of patients and primary care clinicians' views of the key barriers and facilitators to referring to and attending PAT and prescribing/taking penicillin after a negative test. It also highlights the positive effects of the behavioural intervention for both patients and clinicians.

Clinicians who took part in the study were motivated by having access to testing as they were convinced of the benefits of penicillin for their patients and their practice. In line with other studies, clinicians were often unsure about criteria for referral or what allergy testing involves[20,21], and reported little prior experience and only some knew what was involved in the test prior to the trial.

Our survey results also suggested that nurses might be more tentative about referring patients and changing an allergy status on medical records. Studies with hospital nurses suggested that they could play a role in antibiotic stewardship activities, including accurate and detailed documentation

For peer review only - http://bmjopen.bmj.com/site/about/guidelines.xhtml

of penicillin allergy[22,23]. However, more studies are needed to explore the views of this group of healthcare professionals and better define the multidisciplinary approach to de-labelling[24] .

While the baseline survey showed that patients had positive beliefs about testing, including safety, these beliefs seemed to be strengthened after they had completed the PAAP. In line with other studies[23-25], qualitative interviews also highlighted the importance of safety of testing as patients valued having numerous opportunities to address their concerns about safety of the test, including conversations with their GP and later with hospital staff.

Our study identified a number of important clinical implications. First, we note the importance of clinician training through provision of educational materials to ensure successful implementation of referral to PAT and subsequent penicillin prescribing in primary care. The information materials provided were useful to clinicians and increased their knowledge and confidence in referring people and having discussions with patients about PAT. Secondly, it also raises the importance of a clear plan in relation to the de-labelling process, involving clear procedures for informing other colleagues about test results to facilitate penicillin prescribing in the future. Our study indicates that communication between staff member is crucial with dedicated roles being key. Thirdly, while patients may have a number of concerns about safety of PAT[13, 21, 26], these concerns can be successfully addressed and educational materials may be useful in increasing patients' motivation; however, primary care clinicians need to be provided with appropriate resources and time to address these issues.

Strengths and limitations

The study benefits from using both survey and interviews, providing complementary perspectives. Including qualitative studies is important in trial and implementation settings, where they are now recommended as part of the mixed methods evaluations[14]

For peer review only

However, the response rate, and the number of participants in both clinician and patient surveys was low (52.2%; N=26 and 46%; N=53 respectively) thus the results may have to be interpreted with caution, especially as both patients and clinicians taking part in the survey are already motivated to take part in the trial. Our study would have also benefitted from a follow-up survey with clinicians; this was not possible due to study delays. The study recruited from one region in England and includes small numbers due to issues with recruitment and testing availability; hence the results should be extrapolated with caution. Not all trial participants completed the survey, meaning that only views of participants who chose to complete the survey are represented. Both patients and clinician interview participants were a convenience sample recruited within a feasibility study, hence now allowing to capture perspectives of patients and clinicians who did not want to take part in the interviews, which means that these findings may have to be interpreted cautiously in terms of their transferability to other settings.

Conclusions

This study identified a number and issues of which need to be addressed to ensure successful implementation of penicillin allergy de-labelling programmes. However, the study highlighted that both patients and clinicians' concerns can be addressed with help of carefully designed intervention materials.

Acknowledgments: The ALABAMA team consists of: Jenny Boards, Emily Bongard, Christopher C Butler, Mina Davoudianfar, Mandy East, Joanne Fielding, Philip Howard, Sue H Pavitt, Catherine E Porter, Jonathan AT Sandoe, Marta Santillo, Louise Savic, Sinisa Savic, Bethany Shinkins, Sarah Tonkin-Crine, Marta Wanat, Robert West, and Ly-Mee Yu.

For peer review only - http://bmjopen.bmj.com/site/about/guidelines.xhtml

Contributors: Conceptualization, Christopher Butler, Sue Pavitt, Jonathan Sandoe and Sarah Tonkin-Crine; Formal analysis, Marta Wanat, Marta Santillo and Ushma Galal; Funding acquisition, Christopher Butler, Sue Pavitt, Jonathan Sandoe and Sarah Tonkin-Crine; Investigation, Marta Wanat, Marta Santillo, Mina Davoudianfar, Emily Bongard, Sinisa Savic, Louise Savic, Catherine Porter, Joanne Fielding, Christopher Butler, Sue Pavitt, Jonathan Sandoe and Sarah Tonkin-Crine; Methodology, Marta Wanat, Marta Santillo^ and Sarah Tonkin-Crine; Project administration, Catherine Porter and Joanne Fielding; Resources, Sue Pavitt and Jonathan Sandoe; Supervision, Sarah Tonkin-Crine; Writing – original draft, Marta Wanat and Marta Santillo; Writing – review & editing, Ushma Galal, Mina Davoudianfar, Emily Bongard, Louise Savic, Catherine Porter, Joanne Fielding, Christopher Butler, Sue Pavitt, Jonathan Sandoe and Sarah Tonkin-Crine.

Funding: This work was supported by the National Institute for Health Research (NIHR) under its Programme Grants for Applied Research Programme (Grant Number RP-PG-1214-20007) and by the National Institute for Health Research (NIHR) Health Protection Research Unit in Healthcare Associated Infections and Antimicrobial Resistance (NIHR200915) at the University of Oxford in partnership with the UK Health Security Agency (UKHSA) (S.T.-C. and C.C.B.) and by the National Institute for Health Research (NIHR) infrastructure at Leeds. The funders had no role in the design of the study; in the collection, analyses, or interpretation of data; in the writing of the manuscript, or in the decision to publish.

Competing interests: None declared.

Ethics Approval: The study received ethical approval from the London Bridge Research Ethics Committee (Ref: 19/LO/0176). All participants gave written informed consent to take part in the study.

Data availability statement: Survey data are available from authors on reasonable request. Transcripts of interviews are not available as participants have not given permission to share their data.

For peer review only - http://bmjopen.bmj.com/site/about/guidelines.xhtml

References

1. Shenoy ES, Macy E, Rowe T. et al. Evaluation and management of penicillin allergy: a review. *JAMA* 2019; **321:** 188-99.

2. West R, Smith C, Pavitt S. et al. 'Warning: allergic to penicillin': association between penicillin allergy status in 2.3 million NHS general practice electronic health records, antibiotic prescribing and health outcomes. *J Antimicrob Chemother,* 2019; **74:** 2075-82.

3. Minh H-BC, Bousquet PJ, Fontaine C, et al. Systemic reactions during skin tests with [beta]-lactams: a risk factor analysis. *J Allergy Clin Immunol* 2006; **117**: 466.

4. Blumenthal KG, Lu N, Zhang Y. et al. Risk of meticillin resistant Staphylococcus aureus and Clostridium difficile in patients with a documented penicillin allergy: population based matched cohort study. *BMJ* 2018; **361**: k2400.

5. Blumenthal KG, Ryan EE, Li Y. et al. The impact of a reported penicillin allergy on surgical site infection risk. *Clin Infect Dis* 2018; **66**: 329-36.

6. Reddy V, Baman NS, Whitener C et al. Drug resistant infections with methicillin-resistant Staphylococcus aureus, Clostridium difficile, and vancomycin resistant Enterococcus are associated with a higher prevalence of penicillin allergy. *J Allergy Clin Immunol* 2013: **131**: AB170.

7. Macy E, & Contreras R. Health care use and serious infection prevalence associated with penicillin "allergy" in hospitalized patients: a cohort study. *J Allergy Clin Immunol* 2014; **133:** 790-96.

8. Charneski L, Deshpande G, Smith et al. Impact of an antimicrobial allergy label in the medical record on clinical outcomes in hospitalized patients. *Pharmacotherapy: J Human Pharm Drug Ther* 2011; **31:** 742-47.

9. Vyles D, Chiu A, Simpson P, Nimmer M, Adams J, Brousseau D. Parent-reported penicillin allergy symptoms in the pediatric emergency department. *Acad Pediatr* 2017; **17:**251-255.

For peer review only - http://bmjopen.bmj.com/site/about/guidelines.xhtml

10. Gerace K, Karlin E, McKinnon E, Phillips E. Varying penicillin allergy testing practices in the United States: A time for consensus. *J Allergy Clin Immunol* 2015;**3**: 791-793.

11. Trubiano JA, Beekmann SE, Worth LJ et al. Improving antimicrobial stewardship by antibiotic allergy delabeling: evaluation of knowledge, attitude, and practices throughout the emerging infections network. *Open Forum Infect Dis* 2016; **3:** ofw153.

12. Gerace K. & Phillips E. Penicillin allergy label persists despite negative testing. *J Allergy Clin Immunol* 2015; **3:** 815-816.

13. Wanat M, Anthierens S, Butler CC et al. Patient and primary care physician perceptions of penicillin allergy testing and subsequent use of penicillin-containing antibiotics: a qualitative study. *J Allergy Clin Immunol Prac* 2019; **7:** 1888-93. e1.

14. Moore G, Audrey S, Barker M, Bond L, Bonell C, Hardeman W, et al. Process evaluation of complex interventions: Medical Research Council guidance. 2014 Presented at: MRC Population Health Science Research Network; 2014; London URL: http://decipher.uk.net/wp-content/uploads/2014/11/MRC-PHSRN-Process-evaluation-guidance.pdf

15. Yardley, L., Morrison, L., Bradbury, K., & Muller, I. (2015). The person-based approach to intervention development: application to digital health-related behavior change interventions. Journal of medical Internet research, **17:** e4055.

16. Santillo M.; Wanat M.; Davoudianfar M. et al. Developing a behavioural intervention package to identify and amend incorrect penicillin allergy records in UK general practice and subsequently change antibiotic use. *BMJ Open* 2020; **10**: e035793. doi: 10.1136/bmjopen-2019-035793.

17. Cane J, O'Connor D, Michie SJIs. Validation of the theoretical domains framework for use in behaviour change and implementation research. *Implement. Sci*. 2012; **7**: 1-17.

For peer review only - http://bmjopen.bmj.com/site/about/guidelines.xhtml

18. Fusch PI, Ness LR. Are we there yet? Data saturation in qualitative research. *Qual Rep 2015,* 20: 1408.

19. Braun V, Clarke V. Using thematic analysis in psychology. Qual Res Psychol 2006, **3**: 77-101.

20. Wanat M, Anthierens S, Butler CC.; et al. Patient and prescriber views of penicillin allergy testing and subsequent antibiotic use: a rapid review. Antibiotics 2018; **7**:71.

21. Hayoun MB, Bourrier T, Pognonec C. et al. Impact du bilan d'allergie aux bêta-lactamines sur les médecins généralistes dans une cohorte de 154 patients. *Revue Française d'Allergologie* 2015; **55**: 333-40.

22. Monsees EA, Tamma PD, Cosgrove SE et al. Integrating bedside nurses into antibiotic stewardship: A practical approach. *Infect Control Hosp Epidemiol* 2019; **40**: 579-84. doi: 10.1017/ice.2018.362 [published Online First: 2019/02/21]

23. Carter, EJ, Greendyke, WG, Furuya, EY et al. Exploring the nurses' role in antibiotic stewardship: a multisite qualitative study of nurses and infection preventionists. Am J Infect Control. 2018; **46:** 492-497.

24. Staicu ML, Vyles D, Shenoy ES et al. Penicillin Allergy Delabeling: A Multidisciplinary Opportunity. *J Allergy Clin Immunol Practice* 2020, **8**:2858-68.e16. doi: https://doi.org/10.1016/j.jaip.2020.04.059

25. Al-Ahmad M, Rodriguez-Bouza TJ. Drug allergy evaluation for betalactam hypersensitivity: Cross-reactivity with cephalosporines, carbapenems and negative predictive value. Asian Pac J *Allergy Immunol* 2018, **36**: 27-31.

26. Phillips EJ, Knowles SR, O'Brien L. et al. The utility of penicillin skin testing in a tertiary care clinic. J *Allergy Clin Immunol* 2002, **109**: S143.

For peer review only - http://bmjopen.bmj.com/site/about/guidelines.xhtml

Appendices

Appendix 1 Clinician Penicillin Allergy Belief questionnaire

Please respond to the statements below using the scale provided. Please give only one answer per statement.

I understand what is involved in penicillin allergy testing.

☐	☐	☐	☐	☐	☐	☐
Strongly Agree	Agree	Slightly Agree	Not Sure	Slightly Disagree	Disagree	Strongly Disagree

I have previous experience of referring patients for penicillin allergy testing.

☐	☐	☐	☐	☐	☐	☐
Strongly Agree	Agree	Slightly Agree	Not Sure	Slightly Disagree	Disagree	Strongly Disagree

I am happy to refer patients for penicillin allergy testing.

☐	☐	☐	☐	☐	☐	☐
Strongly Agree	Agree	Slightly Agree	Not Sure	Slightly Disagree	Disagree	Strongly Disagree

Penicillin allergy testing can benefit my patients.

☐	☐	☐	☐	☐	☐	☐
Strongly Agree	Agree	Slightly Agree	Not Sure	Slightly Disagree	Disagree	Strongly Disagree

Penicillin allergy testing can benefit my practice.

For peer review only

☐ Strongly Agree	☐ Agree	☐ Slightly Agree	☐ Not Sure	☐ Slightly Disagree	☐ Disagree	☐ Strongly Disagree

It is safe to prescribe penicillin after a negative penicillin allergy test.

☐ Strongly Agree	☐ Agree	☐ Slightly Agree	☐ Not Sure	☐ Slightly Disagree	☐ Disagree	☐ Strongly Disagree

I am confident in discussing penicillin allergy test results with my patients.

☐ Strongly Agree	☐ Agree	☐ Slightly Agree	☐ Not Sure	☐ Slightly Disagree	☐ Disagree	☐ Strongly Disagree

I am happy to change patient records based on the results of penicillin allergy testing.

☐ Strongly Agree	☐ Agree	☐ Slightly Agree	☐ Not Sure	☐ Slightly Disagree	☐ Disagree	☐ Strongly Disagree

I would prescribe penicillin, if indicated, to patients with a negative penicillin allergy test result.

☐ Strongly Agree	☐ Agree	☐ Slightly Agree	☐ Not Sure	☐ Slightly Disagree	☐ Disagree	☐ Strongly Disagree

My colleagues support de-labelling of patients with incorrect penicillin allergy status using penicillin allergy testing.

☐ Strongly Agree	☐ Agree	☐ Slightly Agree	☐ Not Sure	☐ Slightly Disagree	☐ Disagree	☐ Strongly Disagree

For peer review only

My patients would be happy to be referred for penicillin allergy testing.

Strongly Agree	Agree	Slightly Agree	Not Sure	Slightly Disagree	Disagree	Strongly Disagree
☐	☐	☐	☐	☐	☐	☐

My patients would be happy to take penicillin following a negative penicillin allergy test.

Strongly Agree	Agree	Slightly Agree	Not Sure	Slightly Disagree	Disagree	Strongly Disagree
☐	☐	☐	☐	☐	☐	☐

Appendix 2 Patient Penicillin Allergy Belief questionnaire

Below statements to be asked to patients with responses given as Likert scale (1-7):

14. Penicillin is often the best treatment for bacterial infections.

Strongly Agree	Agree	Slightly Agree	Not Sure	Slightly Disagree	Disagree	Strongly Disagree
☐	☐	☐	☐	☐	☐	☐

15. Having an incorrect penicillin allergy label can be harmful for patients.

Strongly Agree	Agree	Slightly Agree	Not Sure	Slightly Disagree	Disagree	Strongly Disagree
☐	☐	☐	☐	☐	☐	☐

16. Having a penicillin allergy test is not beneficial for patients themselves.

Strongly Agree	Agree	Slightly Agree	Not Sure	Slightly Disagree	Disagree	Strongly Disagree
☐	☐	☐	☐	☐	☐	☐

For peer review only

17. Most people in the UK who think they are allergic to penicillin are not actually allergic.

Strongly Agree	Agree	Slightly Agree	Not Sure	Slightly Disagree	Disagree	Strongly Disagree
☐	☐	☐	☐	☐	☐	☐

18. I believe that I am allergic to penicillin.

Strongly Agree	Agree	Slightly Agree	Not Sure	Slightly Disagree	Disagree	Strongly Disagree
☐	☐	☐	☐	☐	☐	☐

19. There is a high chance that I would have a serious allergic reaction if I took penicillin.

Strongly Agree	Agree	Slightly Agree	Not Sure	Slightly Disagree	Disagree	Strongly Disagree
☐	☐	☐	☐	☐	☐	☐

20. It is important for me to know whether or not I am allergic to penicillin.

Strongly Agree	Agree	Slightly Agree	Not Sure	Slightly Disagree	Disagree	Strongly Disagree
☐	☐	☐	☐	☐	☐	☐

21. Penicillin allergy testing is safe.

Strongly Agree	Agree	Slightly Agree	Not Sure	Slightly Disagree	Disagree	Strongly Disagree
☐	☐	☐	☐	☐	☐	☐

22. Penicillin allergy testing can say if someone is allergic to penicillin or not.

Strongly Agree	Agree	Slightly Agree	Not Sure	Slightly Disagree	Disagree	Strongly Disagree
☐	☐	☐	☐	☐	☐	☐

23. I would benefit from being able to take penicillin antibiotics safely when needed in future.

For peer review only

☐	☐	☐	☐	☐	☐	☐
Strongly Agree	Agree	Slightly Agree	Not Sure	Slightly Disagree	Disagree	Strongly Disagree

24. I am frightened about having a serious allergic reaction if I take penicillin.

☐	☐	☐	☐	☐	☐	☐
Strongly Agree	Agree	Slightly Agree	Not Sure	Slightly Disagree	Disagree	Strongly Disagree

25. My doctor would be happy to prescribe penicillin if a test showed I was not allergic to penicillin.

☐	☐	☐	☐	☐	☐	☐
Strongly Agree	Agree	Slightly Agree	Not Sure	Slightly Disagree	Disagree	Strongly Disagree

26. I would be happy to take penicillin prescribed by my doctor if a test showed I was not allergic.

☐	☐	☐	☐	☐	☐	☐
Strongly Agree	Agree	Slightly Agree	Not Sure	Slightly Disagree	Disagree	Strongly Disagree

For peer review only

Appendix 3

Clinician Topic Guide

Below is a list of topics to be discussed in this study. The work will remain flexible with respect to participants' agendas. Therefore, we may add new topics as the interviews progress and data collection continues. However, the key topic of clinicians' views and experiences of managing patients with penicillin allergy and participation in the ALABAMA trial will remain the same.

1. Participants' views and experiences of referring patients for penicillin allergy testing (e.g. views of the test, identification of eligible patients)
2. Participants' views and experiences of prescribing antibiotics following a negative allergy test result (e.g. views on prescribing penicillin, discussing test results with patients)

3. Participants' views and experiences of taking part in the trial (e.g. process of recruiting patients, views of trials procedures, views of the trial documents)
4. Participants' views and experiences of intervention materials (e.g. ease of understanding, helpfulness of the documents)

Example questions (additional questions may be added during interviews following the topics above):

The ALABAMA study:

1. Could you tell me why your practice signed up to the ALABAMA study?
   Prompts: Why was the topic of interest? Who was responsible for joining the study? How/when did you first hear about the study?
2. Could you tell me how you found taking part in the ALABAMA study?
   Prompts: To what extent did you feel informed about the study? Did you have any concerns about inviting patients to the study? How did you find the site training? Was there anything you would add or change to the training you received?

Penicillin allergy testing:

3. What do you think about incorrect penicillin allergy records within general practice?
   Prompts: What impact do incorrect allergy records have on patient care? How confident are you about your knowledge of allergies and penicillin allergy testing? Do you have any concerns about allergy testing?
4. How did you feel about referring patients for penicillin allergy testing as part of the trial?
   Prompts: If you referred patients as part of the trial, how did you find the referral of patients? How confident were you in deciding which patients could be referred for a test? Have you encountered any barriers to referral? How did you overcome them? How did you find the Working Instructions on screening and referral of patients? Have you encountered any barriers while using them? Were they easy to find? Were they easy to read? Is there anything you would change about them?
5. How did you find the consultations with patients when they came to be consented for the trial?
   Prompts: What were the most common questions and concerns raised by patients? How confident were you in addressing patients' questions and concerns? Did you use the Working Instructions for these consultations? Have you experienced any barriers when using these

For peer review only

working instructions? Were they easy to find? Were they easy to read? Is there anything about them that you would change?

6. What did you think about the trial materials that you received during the trial?
   Prompts: Was the information you received helpful? Is there anything that should be added to or changed?

7. You received an information pack to help you manage patients who had had a negative allergy test. What did you think of this document?
   Prompts: How were you given the document? Was it helpful? How easy or difficult was it to follow? Is there anything that should be added to or changed? How did you use the Information pack? What did you think of the electronic pop-up about the test results? Is there anything you would change or add to the electronic pop-up?

8. Patients also received information booklets on penicillin allergy testing as part of the trial. What did you think about the patient information provided?
   Prompts: Did you discuss the information with patients in consultations after the test? How did you use the booklet? Is there anything that you would change or add to the patient booklet? Has any patient used the post-intervention card during a consultation about infections after the test? If so, what did you think about the card?

9. How did you receive the allergy test results for your patients?
   Prompts: What did you think about the letters that you received with the patients' test results? Is there anything that you would change or add to that letter? What did you do when the test came back negative/positive? How confident were you in the results? Did you discuss results with the patient? If so when did this happen and how did you explain the results?

10. What was the process of updating medical records?
    Prompts: Have you updated the medical records following negative test result letters? Did you use the working instructions on updating the medical records? How did you find them? Were they easy to find? Were they easy to read? Is there anything that you would change about them? Have you experienced any barriers in updating medical records? How did you overcome them? What did you think of the information about how to update medical records?

11. To what extent did the test result change the way you manage patients now?
    Prompts: Have any patients with infections consulted you following the test? If so, what happened? Have you prescribed penicillin to any patient who had a negative test who has

For peer review only

presented with an infection which needed antibiotics? If so, what did the patient(s) think about taking penicillin?

12. How would you feel about having extended penicillin allergy testing available in the long-term? Prompts: How do you think you would refer to the service? Who do you think it would benefit? Would you have any concerns about continuing to refer patients?

13. Do you have any other thoughts about how the intervention went?

Patient Topic Guide

Below is a list of topics to be discussed in this study. The work will remain flexible with respect to participants' agendas. Therefore, we may add new topics as the interviews progress and data collection continues. However, the key topic of patients' views and experiences of penicillin allergy testing and participation in the trial will remain the same.

1. Participants' views and experiences of undergoing penicillin allergy testing (e.g. what it was like, barriers and facilitators to attending, views about the results, views on taking penicillin when prescribed first line if allergy test was negative)
2. Participants' views and experiences of taking part in the trial (e.g. process of making decisions to take part, views of trials procedures, barriers and facilitators to taking part, views of the trial documents)
3. Participants' views and experiences of intervention materials (ease of understanding, helpfulness of the documents)

Example questions (additional questions may be added following the topics above):

The AlABAMA study:

1. Can you tell me about why you decided to take part in the AlABAMA study?
   Prompts: What did you think when you first heard about the study? Who did you discuss the study with before deciding to take part?

2. What information were you given about the study?
   Prompts: What did you think of the letter and information you were sent about the study?

3. How helpful was this information?
4. Did you have any concerns about taking part?

For peer review only

Penicillin allergy testing:

5. What did you know about penicillin allergy before taking part in the study?
   Prompts: What did you think about having a penicillin allergy? How much did you know about the benefits of taking penicillin compared to other types of antibiotics? How confident are you about what you know now about it? How much did you know about incorrect penicillin allergy labels? How confident are you about what you know now about it?

6. What did you know about penicillin allergy testing before the trial?
   Prompts: What did you think the test was going to involve? What concerns did you have about the test? What do you think about the appointment you had at your general practice before you took the test?

   Prompt: How useful was the appointment? Could you tell me more about any questions or concerns that you discussed during the appointment? How did the GP/nurse answer your questions and concerns?

[For patients in the PAAP intervention arm]

7. Can you tell me what happened when you went for the test in the allergy clinic?
   Prompts: What information were you given before the test? How did you feel about going for the test? Were there any barriers to attending the test? Which health professionals did you speak to about the test? Were there any difficulties in completing the test at the hospital? ow safe did you feel during the test?

8. Were there any difficulties in completing the test at home?
   Prompts: What barriers did you encounter in completing the test at home? How did you overcome them?

9. What happened after your hospital visit?
   Prompts: What were you told to do following the test? Who did you talk to about the test? How confident were in knowing who to contact in case of a reaction at home?

10. How did you feel about the result?
   Prompts: Who did you discuss the result with?

11. [If negative test result] How do you feel about taking penicillin now?
   Prompts: What would you do if your GP wanted to prescribe you an antibiotic? Have you/will you discuss the test result with your GP? Have you been prescribed penicillin? If so, what did you think about taking penicillin?

For peer review only

[For patients in the PAAP intervention arm: Intervention materials]

12. As part of the trial you were provided with a booklet on penicillin allergy testing. What did you think of this booklet?
    Prompts: Was the booklet helpful? How did you use the booklet? Is there anything you would add or change in the booklet?

13. [For patients with negative test result] When you were sent the allergy test result you were given another booklet and a card. What did you think of these?
    Prompts: Was the booklet/card helpful? How did you use the booklet? Do you still have the booklet? Would you use the card in future? Have you used the card with your GP or nurse? Is there anything you would add or change in the booklet and card?

14. Do you have any other thoughts about how the study went? What changes would you recommend for the future trial?

For peer review only

COREQ (COnsolidated criteria for REporting Qualitative research) Checklist

A checklist of items that should be included in reports of qualitative research. You must report the page number in your manuscript where you consider each of the items listed in this checklist. If you have not included this information, either revise your manuscript accordingly before submitting or note N/A.

Topic	Item No.	Guide Questions/Description	Reported on Page No.
**Domain 1: Research team and reflexivity**			
Personal characteristics			
Interviewer/facilitator	1	Which author/s conducted the interview or focus group?	Page 6
Credentials	2	What were the researcher's credentials? E.g. PhD, MD	page 6
Occupation	3	What was their occupation at the time of the study?	page 6
Gender	4	Was the researcher male or female?	page 6
Experience and training	5	What experience or training did the researcher have?	page 6
Relationship with participants			
Relationship established	6	Was a relationship established prior to study commencement?	page 6
Participant knowledge of the interviewer	7	What did the participants know about the researcher? e.g. personal goals, reasons for doing the research	page 6
Interviewer characteristics	8	What characteristics were reported about the inter viewer/facilitator? e.g. Bias, assumptions, reasons and interests in the research topic	page 6
**Domain 2: Study design**			
Theoretical framework			
Methodological orientation and Theory	9	What methodological orientation was stated to underpin the study? e.g. grounded theory, discourse analysis, ethnography, phenomenology, content analysis	page 7
Participant selection			
Sampling	10	How were participants selected? e.g. purposive, convenience, consecutive, snowball	page 7
Method of approach	11	How were participants approached? e.g. face-to-face, telephone, mail, email	page 7
Sample size	12	How many participants were in the study?	page 11
Non-participation	13	How many people refused to participate or dropped out? Reasons?	page 11
Setting			
Setting of data collection	14	Where was the data collected? e.g. home, clinic, workplace	page 6
Presence of non-participants	15	Was anyone else present besides the participants and researchers?	N/A
Description of sample	16	What are the important characteristics of the sample? e.g. demographic data, date	page 11
Data collection			
Interview guide	17	Were questions, prompts, guides provided by the authors? Was it pilot tested?	Appendix
Repeat interviews	18	Were repeat inter views carried out? If yes, how many?	n/a
Audio/visual recording	19	Did the research use audio or visual recording to collect the data?	Page 6
Field notes	20	Were field notes made during and/or after the inter view or focus group?	Page 7
Duration	21	What was the duration of the inter views or focus group?	Page 11
Data saturation	22	Was data saturation discussed?	Page 6
Transcripts returned	23	Were transcripts returned to participants for comment and/or	No

For peer review only - http://bmjopen.bmj.com/site/about/guidelines.xhtml

Topic	Item No.	Guide Questions/Description	Reported on Page No.
		correction?	
**Domain 3: analysis and findings**			
Data analysis			
Number of data coders	24	How many data coders coded the data?	page 7
Description of the coding tree	25	Did authors provide a description of the coding tree?	pages 12-14
Derivation of themes	26	Were themes identified in advance or derived from the data?	page 7
Software	27	What software, if applicable, was used to manage the data?	n/a
Participant checking	28	Did participants provide feedback on the findings?	n/a
Reporting			
Quotations presented	29	Were participant quotations presented to illustrate the themes/findings? Was each quotation identified? e.g. participant number	pages 12-14
Data and findings consistent	30	Was there consistency between the data presented and the findings?	pages 12-14
Clarity of major themes	31	Were major themes clearly presented in the findings?	pages 12-14
Clarity of minor themes	32	Is there a description of diverse cases or discussion of minor themes?	pages 12-14

Developed from: Tong A, Sainsbury P, Craig J. Consolidated criteria for reporting qualitative research (COREQ): a 32-item checklist for interviews and focus groups. *International Journal for Quality in Health Care*. 2007. Volume 19, Number 6: pp. 349 – 357

Once you have completed this checklist, please save a copy and upload it as part of your submission. DO NOT include this checklist as part of the main manuscript document. It must be uploaded as a separate file.

For peer review only
